# *Castanea sativa* Mill. Leaf: UHPLC-HR MS/MS Analysis and Effects on In Vitro Rumen Fermentation and Methanogenesis

**DOI:** 10.3390/molecules27248662

**Published:** 2022-12-07

**Authors:** Marialuisa Formato, Alessandro Vastolo, Simona Piccolella, Serena Calabrò, Monica Isabella Cutrignelli, Christian Zidorn, Severina Pacifico

**Affiliations:** 1 Department of Environmental, Biological and Pharmaceutical Sciences and Technologies, University of Campania ‘Luigi Vanvitelli’, Via Vivaldi 43, 81100 Caserta, Italy; 2 Department of Veterinary Medicine and Animal Production, University of Naples Federico II, Via Federico Delpino 1, 80137 Napoli, Italy; 3 Pharmazeutisches Institut, Abteilung Pharmazeutische Biologie, Christian-Albrechts-Universität zu Kiel, Gutenbergstraße 76, 24118 Kiel, Germany

**Keywords:** *Castanea sativa* Mill., chestnut leaf, hydrolysable tannins (HTs), flavonoids, UHPLC-ESI-Q*q*TOF HR MS/MS analysis, volatile fatty acids, degradability

## Abstract

*Castanea sativa* Mill. (Fagaceae) is a deciduous tree grown for its wood and edible fruits. Chestnut processing produces residues (burs, shells, and leaves) exploitable for their diversity in bioactive compounds in animal nutrition. In fact, plant-specialized metabolites likely act as rumen modifiers. Thus, the recovery of residual plant parts as feed ingredients is an evaluable strategy. In this context, European chestnut leaves from northern Germany have been investigated, proving to be a good source of flavonoids as well as gallo- and ellagitannins. To this purpose, an alcoholic extract was obtained and an untargeted profiling carried out, mainly by means of ultra-high-performance liquid chromatography/high-resolution tandem mass spectrometry (UHPLC-HR MS/MS) techniques. To better unravel the polyphenol constituents, fractionation strategies were employed to obtain a lipophilic fraction and a polar one. This latter was highly responsive to total phenolic and flavonoid content analyses, as well as to antiradical (DPPH^●^ and ABTS^+●^) and reducing activity (PFRAP) assays. The effect of the alcoholic extract and its fractions on rumen liquor was also evaluated in vitro in terms of fermentative parameter changes and impact on methanogenesis. The data acquired confirm that chestnut leaf extract and the fractions therefrom promote an increase in total volatile fatty acids, while decreasing acetate/propionate ratio and CH_4_ production.

## 1. Introduction

The genus *Castanea* (Fagaceae family) consists of twelve species of deciduous trees native to temperate regions of the northern hemisphere. Among them, *Castanea sativa* Mill. (European or sweet chestnut) is the most representative species in Southern Europe, with Italy representing the largest chestnut-producing area, although its worldwide distribution area is very extensive (Figure S1), ranging from Southern Europe and North Africa to North-Western Europe and eastward to Western Asia [1].

*C. sativa* Mill. is of interest for its precious wood and its edible seed (nut), which is composed of the fruit, the pericarp (outer shell), the integument (inner shell), and the surrounding bur. In recent years, the consumption of fresh or processed chestnuts has significantly increased due to their positive effects on health. In fact, chestnut fruits can be used as an energy source due to their content of carbohydrates (especially starch) and fatty acids (mainly linoleic, oleic, and palmitic acids). In addition, chestnuts, as purée and chestnut flour, are finding a new nutritional application as ingredients in gluten-free diets [2]. Indeed, because only the nuts are used for food processing, the shells, leaves, and burs represent common waste products of the chestnut food industry, even if these residual components contain promising bioactives [3]. In this context, recent studies have shown that by-products and waste from chestnut processing are rich in bioactive compounds with antioxidant, antimicrobial, and anti-inflammatory properties that make them good candidates in various fields of application, such as pharmaceuticals, food, or cosmetics [4,5,6,7]. Shells are the most commonly investigated form of chestnut waste, being applicable as a natural antioxidant and prebiotic for human consumption, due to the presence of tannins (condensed and hydrolysable), flavonoids (derivatives of quercetin, rutin, catechin, and apigenin) [8], vitamins E, lignin, and nondigestible oligosaccharides (fiber, cellulose, and hemicellulose) [9,10]. The diversity in bioactive compounds also involves foliar biomass, which is not highly valued since it is considered an agroforestry residue that does not impact the food industry. Exploiting all the non-edible components of the chestnut, including the leaves, enables the production of quality products [11] with a sustainable reduction in environmental pollution in a circular economy scenario. One of the most promising applications is the recovery of forest biomass and chestnut waste (e.g., leaves, shells, burs) for the optimization of feed ingredients as a strategy to achieve sustainable animal production [12,13]. The growing demand for animal products has led to the adoption of intensive production systems that could compromise animal health, affecting the metabolic status and immunological systems of animals. Moreover, this breeding approach is characterized by a higher environmental impact. Thus, there is renewed interest in the use of dietary phytochemicals as natural rumen modifiers, considering the potential concerns related to the use, over the presence, of chemical residues in animal products and the development of bacterial resistance to antibiotics [14]. Indeed, plant material, or its extracts, could be an interesting source for novel food additives in livestock feed [15] as an alternative and natural strategy to ensure animal welfare and product quality. The different nutritional strategies are in tune with the induction of changes in the rumen microbiome, which affects metabolism, productivity, environmental emissions (methane and nitrogen), and the quality of food derived from ruminants. It is known that the integration of polyphenols into the diets of ruminants can modulate the diversity and activity of rumen microorganisms, the degradability of nutrients, and the methanogenesis of the rumen, with effects on the final fermentation products (i.e., N-NH_3_, pH, acetate, butyrate, propionate, etc.). In this regard, the mainly hydrolysable chestnut tannins (HTs) have in some cases shown inhibitory effects on *Ruminococcus flavefaciens*, *Butyrivibrio proteoclasticus*, methanogenic microbiome, and the increase in *B. fibrisolvens,* and in other cases have shown no effect on the microbial community of the rumen [16,17,18,19]. Different types of tannins (HTs vs. condensed tannins, CTs), as well as flavonoids, can have controversial effects on rumen fermentation and microbiome, so much so that a careful chemical analysis is mandatory. It has been further observed that condensed tannins, due to their difficult hydrolysis, exert a greater effect on nutritional profile and digestibility than hydrolysable ones [20].

Herein, renewable *Castanea sativa* Mill. leaves were of interest, and the alcoholic extract obtained by maceration was first investigated through an untargeted UHPLC-HRMS/MS approach. Furthermore, the extract underwent fractionation, obtaining fractions variously enriched in bioactives, as confirmed by a preliminary screening of total phenol content (TPC) and total flavonoid content (TFC). The antiradical and reducing activity of the alcoholic extract and fractions therefrom was also evaluated by means of DPPH^●^ and ABTS^●+^ tests, and by ferricyanide FRAP assay. The further chemical investigation of fractions was carried out by means of UV-Vis spectroscopy and ultra-high-performance liquid chromatography coupled to quadrupole time-of-flight tandem mass spectrometry (UHPLC-Q*q*TOF-MS/MS) analyses. Because the bioactivity assessment is closely coupled to chemical composition, all the differently constituted fractions were tested to evaluate their effects on in vitro ruminal fermentation (cumulative gas production, organic matter degradability, fermentation kinetics, pH, and end products), including methanogenesis.

## 2. Results and Discussion

**Figure 1:**
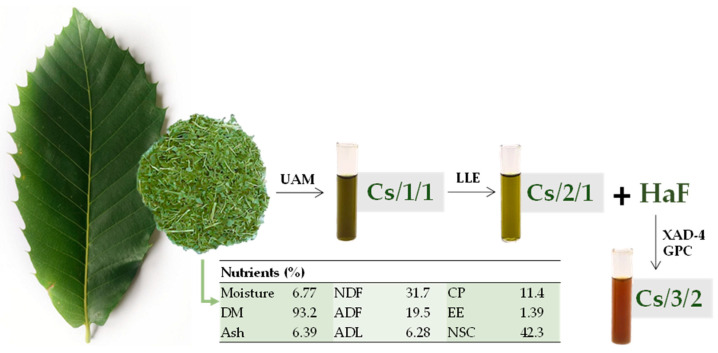
*Castanea sativa* Mill. leaf chemical composition parameters expressed as % of DM, and fractionation scheme to achieve bioactive fractions. DM: dry matter; NDF: neutral detergent fiber; ADF: acid detergent fiber; ADL: acid detergent lignin; CP: crude protein; EE: ether extract; NSC: no structural carbohydrates; UAM: ultrasound assisted maceration; LLE: liquid–liquid extraction; HaF: hydroalcoholic fraction; XAD-4 GPC: gel permeation chromatography on Amberlite XAD-4 absorbent resin.

Leaves of *C. sativa* were preliminarily investigated in terms of dry matter (DM), ash, neutral (NDF) and acid (ADF) detergent fiber, acid detergent lignin (ADL), ether extract (EE), non-structural carbohydrates (NSC), and crude protein (CP). Data acquired are reported in Figure 1 as % of dry matter (DM). In particular, it was observed that NDF and ADF were 31.7 and 19.5% of DM, respectively, whereas the acid detergent lignin (ADL) was equal to 6.28% of DM. The NDF and ADF contents in chestnut leaves, which provide the level of structural carbohydrates, define the good quality of the chestnut biomass and its capability to ensure adequate amounts of fiber and provide a valuable source of energy. Indeed, as is well-known, NDF and ADF values vary widely, based on soil type and other environmental factors, which affect lignin and hemicellulose content and nutrient digestibility [21]. The low crude protein value instead suggests the need to properly balance the dietary composition. In the investigation of the use of chestnut leaves for bioactive compounds, leaves previously collected in Northern Germany were exsiccated and pulverized and subjected to alcoholic ultrasound-assisted maceration. The alcoholic extract obtained, hereafter Cs/1/1, was subjected to fractionation to achieve a non-polar fraction Cs/2/1 and a hydroalcoholic one, which further provided the alcoholic fraction Cs/3/2.

**Figure 2:**
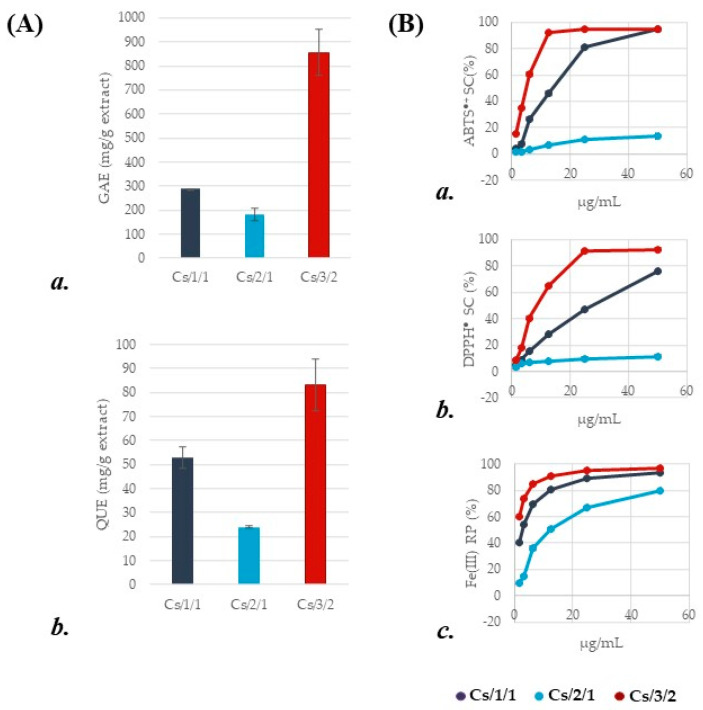
(**A**) (**a**) Total phenolic content (TPC), expressed as mg of gallic acid equivalents (GAEs) per g of extract; (**b**) total flavonoid content (TFC), expressed as mg of quercetin equivalents (QUEs) per g of extract. Values reported are the mean ± SD of three independent measurements. (**B**) (**a**) Scavenging capability (SC%) vs. 2,2′-azino-bis(3-ethylbenzothiazoline)-6-sulfonic acid (ABTS) radical cation; (**b**) scavenging capability (SC%) vs. 2,2-diphenyl-1-picrylhydrazy (DPPH) radical, and (**c**) Fe(III) reducing power (RP%) of *C. sativa* extract and organic fractions therefrom. Values reported are the mean ± SD of three independent measurements.

The chemical composition assessment, preliminarily carried out using colorimetric assays, markedly differentiated Cs/1/1 and its fractions Cs/2/1 and Cs/3/2. The first one showed TPC and TFC values equal to 288.1 ± 1.1 gallic acid equivalents (GAEs) and 52.9 ± 4.4 mg quercetin equivalents (QUEs) per g of extract, respectively (Figure 2A(a,b)). These values are in line with those by Cerulli et al. [22], who, evaluating the TPC and TFC of a chestnut leaf methanol extract, found that total phenols were 299.0 ± 14.8 mg GAEs, while flavonoids were 45.54 ± 0.99 mg RUEs per g of extract. Indeed, due to fractionation, Cs/3/2 appeared to be enriched in phenolic and flavonoid compounds, exhibiting a TPC value threefold higher than Cs/1/1, while flavonoids accounted for 83.2 ± 10.7 mg QUEs per g of extract. To clarify the main actors of Cs/3/2 TP content, which could be attributable to gallo- and ellagitannins [23,24], UHPLC-ESI-QqTOF tandem mass spectrometry analysis was carried out, as well as antiradical and reducing power assessment. In this latter regard, Cs/3/2 showed marked scavenging activity with both ABTS^●+^ and DPPH^●^, with relative ID_50_ values equal to 11.2 ± 0.3 µg/mL and 30.3 ± 0.8 µg/mL. Moreover, Cs/3/2 effectively reduced ferric ions at the lowest tested doses, so much so that ID_50_ and TEAC (Trolox Equivalents Antioxidant Capacity) values were 0.37 ± 0.02 µg/mL and 4.7, respectively (Figure 2B(a–c)).

### 2.1. Chemical Investigation of Alcoholic Leaf Extract and Cs/2/1 and Cs/3/2 Fractions

**Figure 3:**
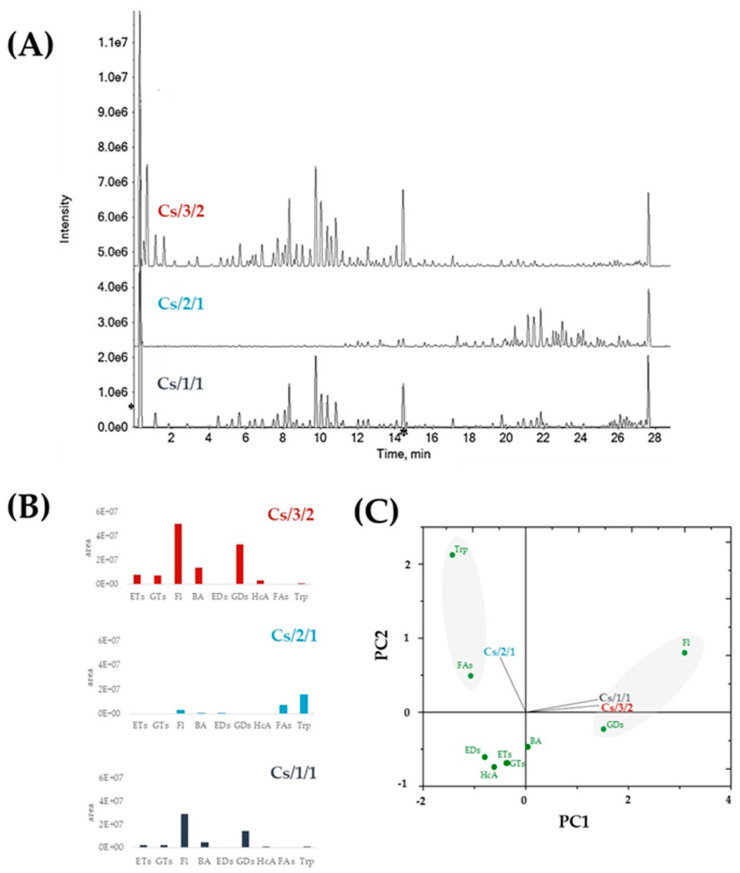
(**A**) Total ion current (TIC) chromatograms of Cs/1/1 extract, Cs/2/1 fraction, and Cs/3/2 fraction. (**B**) The relative content of each extract/fraction in derivatives of benzoic acid (BA), hydroxycinnamic acid (HcA), derivatives of gallic (GDs) and ellagic acid (EDs) as well as flavonoids (Fl), gallotannins (GTs), ellagitannins (ETs), fatty acids (FAs), and triterpenes (Trp), is shown. (**C**) Principal component analysis (PCA), based on different classes of identified compounds. PC1 % variance = 68.3; PC2 % variance = 31.3.

According to literature data, *C. sativa* leaf extracts, obtained using different alcoholic or hydroalcoholic extractants, are a promising source of natural antioxidants [25,26]. The bioactivity of chestnut leaf extracts is involved in suppressing the Nrf2-mediated antioxidant system in cancer stem cells through ROS accumulation and apoptosis-inducing effects [25], as well as by attenuating TLR2- and TLR4-induced inflammatory responses [26]. These and other properties made chestnut leaves usable in topical applications for preventing and/or treating oxidative stress-mediated diseases and photo-ageing [6,27,28,29]. In addition, *Castanea sativa* leaf showed antimicrobial activity against such multiresistant bacteria as *Staphylococcus epidermidis*, *S. aureus*, and *Pseudomonas aeruginosa* [30]. Herein, in order to hypothesize the use of this forest residue in ruminant feeding, the chemical composition in terms of specialized metabolites was tentatively determined by means of UHPLC-HR MS/MS analyses. These latter, firstly carried out on the alcoholic chestnut leaf extract, highlighted the occurrence of ellagitannins, and flavonoid mono- and di-glycosides, beyond fatty acids and terpene compounds. When fractionation was carried out, tannins and flavonoids appeared to constitute Cs/3/2. This was confirmed by the UV-Vis spectrum of the fraction (Figure S2, Supplementary Material), whose bands at 365, 267, and 215 nm were in line with flavonoid and gallo- and ellagitannin electronic transitions [31]. Different spectroscopic and spectrometric behavior was noted for Cs/2/1 fraction. In fact, the UV-Vis absorption bands occurred at 667 and 415, attributable to chlorophylls and carotenoids, and at 236 and 205 nm. These latter are commonly ascribed to fatty acids and their oxidative derivatives, as well as to triterpenoids. Indeed, the mass spectra of Cs/2/1 compounds ascertained a complexity greater than that of Cs/1/1, due to the triterpenes, acylated flavonoids, and ellagic acid methyl derivatives. In Table 1, Table 2, Table 3 and Table 4, as well as Table S1, data from UHPLC-Q*q*TOF-MS/MS analyses on all three extracts are reported. The relative quantification assessment was also performed and plotted using principal component analysis (PCA; Figure 3C).

#### 2.1.1. Chestnut Leaf Tannins and Beyond

Among the polar minor constituents of the chestnut alcoholic extract (Table 1 and Table 2), the greater part was found only in the Cs/3/2 fraction together with gallo- and ellagitannins.

Quinic acid (**1**) and a dihydroxybenzoic acid hexoside ([M-H]^−^ at *m*/*z* 315.0724; **10**; Figure S3, Supplementary Material) were mainly found together with gallic acid (**5**), hexahydroxydiphenic acid (**4**; Figure S4, Supplementary Material) with deprotonated molecular ion at *m*/*z* 337.02, and its decarboxylated derivative with [M-H]^−^ at *m*/*z* 293.03 (3). Three galloyl hexose isomers (**2**, **6**, **12**; Figure S5, Supplementary Material; Table 2), mainly differing in the glycosylation site, the digalloyl hexoses **7** and **30** (Figure S6), and digalloyl deoxyhexoside (**38**) with [M-H]^−^ ion at *m*/*z* 467.0843 were tentatively identified. Tri- and tetragalloyl hexosides were also recognized (**33**, **43**, and **47**). Galloyl moiety was identified based on the characteristic loss of 152.01 Da and fragment ions at *m*/*z* 169.01 and 125.02, and radical ions at *m*/*z* 124.01. It was found in galloyl shikimic acids (**14**, **16**, and **18**), which shared the [M-H]^−^ ion at *m*/*z* 325.05 and the loss of 156.05 Da, as well as in galloyl dihydroxybenzoic acid (**17**; Figure S7, Supplementary Material). Galloyl benzyl alcohol (**8** and **11**) and phenol (**40** and **42**) hexosides, previously identified in chestnut and reported as crenatin and cretanin isomers [32], were also tentatively identified (Figure S8, Supplementary Material). Chestanin (**49**) and isochestanin (**58**), both with the [M-H]^−^ ion at *m*/*z* 937.19, were identified. The TOF-MS/MS spectra of these compounds differed for the fragment ion at *m*/*z* 637.1067, detectable only for compound **49**, which was from the loss of the terminal trihydroxy benzyl hexose unit (Figure S9, Supplementary Material). Among the hydroxycinnamoyl based compounds (Table 1), 5-*O*-caffeoyl quinic acid (**26**), and *p*-coumaroyl quinic acid isomers (**35** and **36**) were detected, as well as the hexosides of *p*-coumaric acid (**20** and **22**) and caffeic acid (**24**) [33].

Ellagitannins (ETs) and flavonoids accounted for a large part of Cs/3/2. Indeed, although some acylated flavonoids derivatives were also found as constituents of the apolar Cs/2/1 fraction, ellagitannins were only found in Cs/3/2 in higher amounts than in Cs/1/1 (Figure 3B; Table 2). The negative UHPLC-HRMS spectra of these compounds mostly showed, other than the deprotonated molecular ion, the doubly charged molecular ion [M-2H]^2−^. Highly diagnostic fragment ions, due to neutral losses of ellagic acid or hexahydroxydiphenoyl moiety (302 Da), galloyl moiety (152 Da), galloyl-hexose (332 Da), HHDP-hexose (482 Da), and galloyl-HHDP-hexose (634 Da), allowed ETs to be putatively identified [31]. The hexahydroxydiphenoyl (HHDP) residue, whose intra-molecular esterification reaction provided ellagic acid, was the main chemical feature of most of ETs monomers. This residue can oxidize, forming the dehydrohexahydroxydiphenoyl (DHHDP) residue, which in turn gives rise to the chebuloyl moiety. Furthermore, the coupling of HHDP with another galloyl residue provides the nonahydroxytriphenoyl (NHTP) group, characteristic of *C*-glycosidic ETs with an opened hexose core [31]. Herein, different ETs, also sharing chebuloyl or valenoyl groups, were tentatively identified. Compounds **9** and **15**, with [M-2H]^2−^ ion at *m*/*z* 466.0328(6), in accordance with the molecular formula C_41_H_26_O_26_, were tentatively identified as NHTP-HHDP-hexose isomers (e.g., castalagin/vescalagin). These compounds were previously found as constituents of an antioxidant and cardioprotective alcoholic extract from *Castanea sativa* Mill. burk, together with ellagic acid and other minor ETs [34]. Compounds **28** and **31**, with the [M-H]^−^ ion at *m*/*z* 935.0818(01), as well as compound **41**, were tentatively galloyl-bis-HHDP-hexose. The TOF-MS/MS spectra showed the consecutive losses of H_2_O and CO_2_, providing fragment ions at *m*/*z* 917.07 and 873.08, respectively, whereas the losses of 152 Da (galloyl moiety) and 302 Da (HHDP group) led to fragment ions at *m*/*z* 783.07 and 633.07, respectively.

**Figure 4:**
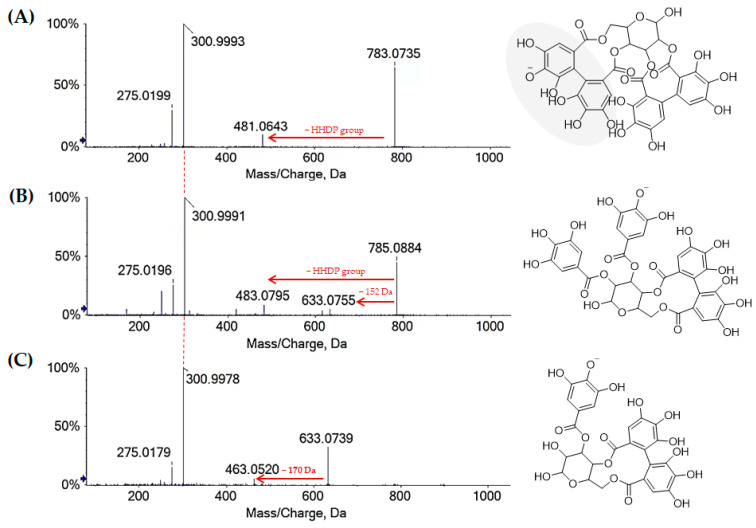
TOF-MS/MS spectra of ellagitannins **13** (**A**), **25** (**B**) and **27** (**C**) with [M-H]^−^ ions at *m*/*z* 783.0735, 785.0884 and 633.0739, which were detected in alcoholic extract Cs/1/1 and its polyphenols enriched extract Cs/3/2.

Compounds **21** and **32** were likely derivatives of compounds **28** and **31** through a putative HHDP oxidation. In fact, TOF MS/MS spectra of both the isomers highlighted the loss of a chebuloyl moiety to achieve, from the deprotonated molecular ion at *m*/*z* 953.09, the ions at *m*/*z* 785.08 and 633.07, respectively. Furthermore, compound **32** showed the [M-H-H_2_O]^−^ and [M-H-CO_2_]^−^ ions at *m*/*z* 935.0828 and 909.1031, while only the decarboxylated ion was found for compound **21**, possibly due to a different accessibility of hydroxyl group. Bis-HHDP-hexose isomers ([M-H]^−^ at *m*/*z* 783.07) were compounds **13** and **19**, most likely casuariin or pedunculagin isomer. Additionally in this case, the loss of HHDP-group (302 Da) provided the ion [M-H-HHDP]^−^ at *m*/*z* 481.0624(43), corresponding to HHDP-hexose structure (Figure 4A). A key reaction of ellagitannins is the release of the bislactone and formation of a HHDP ester group, which undergoes rapid lactonization to yield ellagic acid.

Compounds **25** and **37** were tentatively identified as digalloyl-HHDP-hexoses (Figure 4B). In the TOF-MS/MS spectra of both the compounds, the typical losses of galloyl, gallic acid, and HHDP group were observed within fragment ions at *m*/*z* 633.0775, 615.0650, and 483.0795 for compound **25**, and 633.0755, 615.0645, and 483.0799 for compound **37**. Compound **27** ([M-H]^−^ at *m*/*z* 633.0742) was tentatively identified as galloyl-HHDP-hexose (Figure 4C).

The TOF-MS/MS spectrum of the compound **23** ([M-H]^−^ at *m*/*z* 951.0761) suggested HHDP-valoneoyl-hexose occurrence (Figure S10). The 168 Da loss provided the fragment ion at *m*/*z* 783.0708. This loss could be attributable to the decarboxylation of the [M-H]^−^ ion to give the [M-H-CO_2_]^−^ ion at *m*/*z* 907.0857, which further lost 124 Da. When the [M-H-CO_2_]^−^ ion lost 302 Da, the ion at *m*/*z* 605.0796 was formed, whereas the loss of the HHDP residue from the ion at *m*/*z* 783.0708 supplied the ion at *m*/*z* 481.0693. Methylvaloneoyl-NHTP-hexose was also recognized (**29**); the ions at *m*/*z* 933.0706 and 631.0572 were in line with [M-H-C_8_H_6_O_5_]^−^ and [M-H-C_22_H_14_O_13_]^−^, whereas methylation on valoneoyl group was also confirmed by the base peak at *m*/*z* 181.0132. Instead, compound 44 with [M-2H]^2−^ ion at *m*/*z* 552.0490 was tentatively identified as tri-galloyl-valoneoyl-HHDP-hexose (e.g., rugosin A). The structural hypothesis was confirmed by the TOF-MS/MS spectrum, which, beyond the characteristic losses of 152 (galloyl), 170 (gallic acid), and 302 Da (HHDP), showed the loss of 168.00 Da from the deprotonated molecular ion to provide the fragment ion at *m*/*z* 937.1942.

Chesnatin (**34**) was differentiable from isochesnatin (**39**), while compound **45** was tentatively identified as trigalloyl-HHDP-hexose. Furthermore, compound **48** with [M-H]^−^ at *m*/*z* 433.0415 was likely an ellagic acid pentoside; TOF-MS/MS spectrum showed the neutral loss of dehydrated pentose providing fragment ions at *m*/*z* 300.9979 and 299.9902, diagnostic for ellagic acid. In the apolar fraction Cs/2/1, trimethylellagic acid isomers (**82** and **87**) and their hexosyl (**69**) or deoxyhexosyl (**81**) derivatives occurred. Trimethylellagic acid glycosides were detected in the TOF-MS spectra as formic acid adducts with [M+HCOOH]^−^ ion at *m*/*z* 551.1057 (**69**) and 535.1106 (**81**) for the lack of a primarily acid site being all phenolic hydroxy groups methylated or involved in the glycosidic bond.

#### 2.1.2. Chestnut Leaf Flavonoids

**Figure 5:**
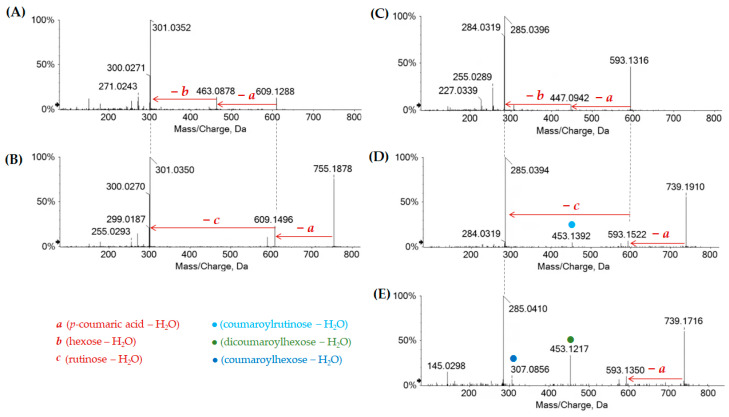
TOF-MS/MS spectra of the acylated quercetin glycosides **75** (**A**) and **76** (**B**) and acylated kaempferol glycosides **77** (**C**), **86** (**D**) and **92** (**E**), which were detected also in the apolar fraction Cs/2/1.

Flavonoids in the investigated chestnut leaf extract and derived fractions are listed in Table 3. Quercetin (**71**, [M-H]^−^ ion at *m*/*z* 301.0354), kaempferol (**80**, [M-H]^−^ ion at *m*/*z* 285.0403), isorhamnetin, and methylkaempferol (**89**, [M-H]^−^ ion at *m*/*z* 299.0559) were found as the most abundant flavonol aglycone cores. Flavones, such as luteolin (**72**, [M-H]^−^ ion at *m*/*z* 285.0398), and acacetin (**93**, [M-H]^−^ ion *m*/*z* 283.0611), were also tentatively identified as constituents of Cs/2/1 and Cs/3/2 fractions. The discrimination between kaempferol and luteolin was based on their relative retention time and the presence/absence of the luteolin diagnostic fragment ion at *m*/*z* 133.0297. Quercetin pentosyl-hexoside (**46**), quercetin hexuronides (**50** and **54**), quercetin 3-*O*-hexosides (**51**, **53**, and **55**), quercetin 3-*O*-pentoside (**57**) and rutin (**52**) were tentatively identified. Neutral losses of 294.09 (dehydrated hexose + pentose), 176.03 Da (dehydrated hexuronic acid), 162.05 Da (dehydrated hexose), 132.04 Da (dehydrated pentose), and 308.11 (dehydrated hexose + deoxyhexose) suggested the glyconic moiety identity. Different mono- (**61**, **64–66**, and **68**) and diglycosidic (**62** and **67**) derivatives of isorhamnetin were also identified. TOF-MS/MS spectra of compounds showed the classic neutral losses of hexose, deoxyhexose (−146.05 Da), hexuronidyl, and deoxyhexosyl-hexose, providing the [aglycone-H]^−^ and [aglycone-H]^●+^ ions at *m*/*z* 315.05 and 314.04, respectively, from which the loss of 15 Da occurred, generating fragment ions at *m*/*z* 300.02 and 299.0195. Quercetin and kaempferol acylated derivatives were also recognized in fraction Cs/2/1. Metabolite **75** ([M-H]^−^ ion at *m*/*z* 609.1258) and metabolites **76** and **79** ([M-H]^−^ ion at *m*/*z* 755.1850(4)) were tentatively identified as quercetin *p*-coumaroyl-hexoside and quercetin *p*-coumaroyl deoxyhexosyl hexoside, respectively (Figure 5A,B). Furthermore, mono- and di-*p*-coumaroyl kaempferol glycosides were also detected (**77**, **83**, **85**, **86**, **91** and **92**) (Figure 5C–E).

Acyl glycosides of quercetin and kaempferol were putatively identified through the neutral losses of 308.09 Da (*p*-coumaroylhexose-H_2_O), 454.15 Da (*p*-coumaroyldeoxyhexosylhexose-H_2_O), and 454.13 Da (di-*p*-coumaroylhexose-H_2_O). The TOF-MS/MS spectrum of compound **78** ([M-H]^−^ at *m*/*z* 533.1316) was in accordance with the butyl-ester of quercetin hexuronide. In fact, the loss of dehydrated glucuronic acid (−176 Da) and the neutral distinctive loss for butyl group (−56 Da) were observed. This compound was also identified in *Gaultheria procumbens* L. [35] and *Vitis vinifera* L. leaf [36], showing an inhibition and synergic effect in combination with clarithromycin against *Helicobacter pylori*. Among the kaempferol derivatives, two kaempferol deoxyhexosyl-hexosides (**60** and **63**) and acetylhexosides (**70** and **73**) were also tentatively identified, together with mono- and diacetylated kaempferol *p*-coumaroylhexoside and di-*p*-coumaroylhexoside (**88**, **96**, **97**, **100** and **102**), which were only in the apolar Cs/2/1.

#### 2.1.3. Chestnut Leaf Lipids

Beyond flavonoids, the alcoholic extract and mainly its fraction Cs/2/1 contained fatty acids and terpenoids (Table 4). Among fatty acids, there were dodecanedioic acid (**84**, [M-H]^−^ at *m*/*z* 227.1291), likely traumatic acid, 9-oxooctadeca-10,12-dienoic acid (**106**, [M-H]^−^ at *m*/*z* 293.2123) and 9-oxooctadeca-10,12,15 trienoic acid (**108**, [M-H]^−^ at *m*/*z* 291.1965). These latter were previously identified in *F. sylvatica* L. apolar leaf fraction [15]. Tri- and di- unsaturated C18 fatty acids were also recognized as linolenic (**116**) and linoleic (**119**) acids, respectively. Furthermore, a glycerolipid (**105**) and glycerophospholipids (**109** and **111**) were also detected [22,37]. Compounds **109** and **111** were tentatively identified as *lyso*-PA (18:3) and *lyso*-PA (16:0), respectively for low intensities of their linked fatty acids [38].

Furthermore, fraction Cs/2/1 accounted for a substantial triterpene component. According to literature data, different cycloartane-type triterpenoids were found in *Castanea cornata* burs [39], and ursane and oleanane derivatives were found in *C. sativa* leaf [40]. The [M+HCOOH]^−^ ion for the compound **90** at *m*/*z* 695.4037 was in accordance with the 3,7,24-trihydroxy-cycloartene-28-oic acid hexoside [22,39]. The TOF-MS/MS spectrum displayed the ion at *m*/*z* 487.3450 (formed by the loss of 162.05 Da from the detectable deprotonated molecular ion, with a theoretical *m*/*z* value of 649.3972), which gave the fragment ion at *m*/*z* 469.3326 and 441.3377 through the relative losses of 18 Da and 44 Da.

Compound **94**, with [M-H]^−^ ion at *m*/*z* 461.2921 and molecular formula C_27_H_42_O_6_, was tentatively identified as 3,7-dihydroxynorcycloartane 24,28-dioic acid [39]. The consecutive losses of H_2_O (18 Da) and CO_2_ (44 Da) from the deprotonated molecular ion provided fragment ions at *m*/*z* 443.2808 and 397.2786, whereas the direct loss of carbon dioxide provided fragment ion at *m*/*z* 415.2846. Indeed, compound **98** was likely a derivative of the previous compound, in which aldehydic group was instead of carboxylic one in the chain. Compounds **99**, **101,** and **103**, with [M-H]^−^ ion at *m*/*z* 487.34 could be also cycloartane-type terpenoids, while compound **98** was putatively identified as a hydroxyl derivative of compound **95**. Recently, castaartancrenoic acids D and E, sharing the molecular formula C_30_H_47_O_5,_ were isolated from the burs of *Castanea crenata* [39]. Compounds **104**, **107**, **111**, **112**–**115**, and **117** were likely ursane/oleanane type triterpenoids [40]. Recently, two new ursane-type triterpenoid saponins named chestnoside A and chestnoside B were identified [41]; however, little information is available in the literature on the isolation and identification of these metabolites in *Castanea sativa* Mill.

### 2.2. Effects of Chestnut Leaf Alcoholic Extract and Its Fractions on In Vitro Rumen Fermentative Activity

The use of bioactive compounds as natural feed supplements has been extensively studied as a strategy to manipulate rumen fermentative activities, and the effects of flavonoid and tannin extract as supplements in animal nutrition have been increasingly investigated, especially for ruminants. The availability of literature for condensed tannins (CTs) is extensive, whereas hydrolysable tannins (HTs) have been less extensively explored in animal nutrition, even if little attention is given to the chemical composition of the treated extracts. Data about the rumen fermentation effects of *Castanea sativa* extracts leaf and/or only leaves are scarce [42]; however, different studies reported the effects of chestnut involucre or tannin wood extracts on ruminal fermentation [13,43,44,45].

**Figure 6:**
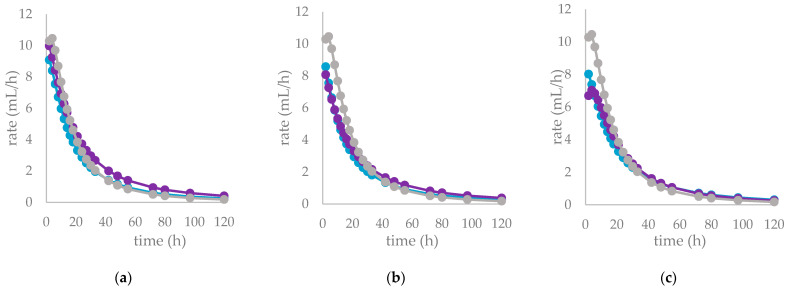
In vitro fermentation rate over time of *C. sativa* Cs/1/1 extract (**A**) and its fractions Cs/2/1 (**B**) and Cs/3/2 (**C**) at the 50-mg (●) and 200-mg (●) dose levels and control diet (●).

Nonetheless, herein, the presence of other bioactive compounds, such as flavonoids, triterpenes, or fatty acids, in the Cs/1/1 and Cs/3/2 fractions might interfere and modulate the tannin effects. Tannins generally exert a dual mechanism: high doses reduce voluntary feed intake and nutrient digestibility, whereas moderate concentrations can improve nutrient utilization. Moreover, tannins modify the digestive processes of ruminants not only by binding dietary protein, but also through modulation of rumen microbiota; not surprisingly, hydrolysable and condensed tannins as well as flavonoids are considered as antimicrobial feed additives, due to their antibacterial and antiparasitic activity [46]. Furthermore, these secondary metabolites can inhibit the enzymatic activity of microbial protease and urease into the rumen [47]. The supplementation of polyphenols in ruminant diet can modulate the diversity and activity of rumen microorganisms, the nutrient degradability and rumen methanogenesis. The differences between substrates in the fermentation process are clearly illustrated in Table 5 and Figure 6, where the gas production rate and in vitro fermentation rate over time are shown. The organic matter degradability (OMD) after 120 h of incubation showed, for the all-chestnut-based diet, values lower than the control diet. It was observed that chestnut tannins linearly decreased OMD [48] as well as *Castanea sativa* leaves [42]. Analyzing the fermentation effects of tannic acid (HT) and quebracho tannins (CTs) on wheat and corn grain, Martìnez et al. [49] highlighted a reduction in gas production after 24 h of incubation by means of a physical mechanism. Scanning electron microscopy (SEM) revealed that both sources of tannins slowed starch hydrolysis through degradation of the endosperm protein matrix. Due to their chemical structure, tannins can bind protein through hydrogen bonds forming tannin–protein complexes that are stable at the rumen pH (5.0–7.0) [50]. Similarly, except for Cs/1/1 at the 200-mg dose level, the tested samples produced a lower amount of gas (OMCV) than the control diet. In particular, the lipophilic fraction Cs/2/1 exhibited the lowest values (*p* < 0.001) for OMD and OMCV at both tested dose levels. Furthermore, the Cs/2/1-200 mg sample showed the lowest value for T_max_ and a significative decrease (*p* < 0.05) for R_max_. Beyond the Cs/2/1-200 mg dose level, for the Cs/3/2-50 mg sample, a reduction in both parameters T_max_ and R_max_ was also noted.

**Figure 7:**
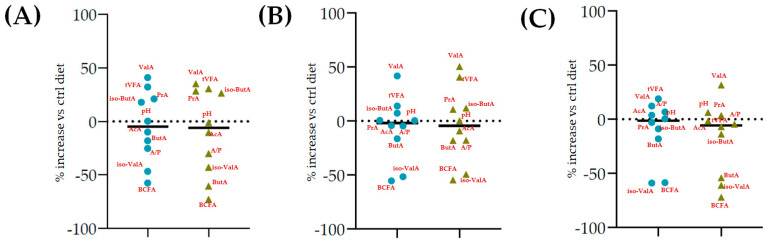
Scatter plots of the percentage increase or decrease in each volatile fatty acid and pH after 24 of incubation for different tested dose levels (● 50 mg and ▲ 200 mg) vs. % in the control diet, considering Cs/1/1 extract (**A**) and its fractions Cs/2/1 (**B**) and Cs/3/2 (**C**).

The fermentation rates after 24 h of incubation following the different *C. sativa* extract supplementations are reported in Table 6, while data acquired in terms of pH and the relative concentration of the fermentation end products are listed in Table S2 and represented by the scatter plot in Figure 7. In vitro fermentation end products after 120 h of incubation were reported in Table 7, and according to the single volatile fatty acids, are reported as proportion (%) of single volatile fatty acids within the total volatile fatty acid (VFAs) content, expressed as mmol/L (Table 7).

The results after 24 h of incubation showed that supplementation with the extract does not depress degradability; even the 200 mg dose levels of three extracts seemed to significantly improve organic matter degradability (OMD). The rumen pH did not appear to change after 24 h. By contrast, Hassanat and Benchaar [43] demonstrated a significant increase in rumen pH after only 24 h of incubation with different tannin extracts sources (i.e., chestnuts, acacia, quebracho, and valonia). In our case, after 120 h, the rumen pH was significantly modified by the chestnut alcoholic extract and its fractions. In particular, the Cs/2/1-50 mg sample showed the highest value of rumen pH followed by Cs/1/1-50 mg and Cs/1/1-200 mg samples. Only the Cs/3/2-200 mg sample showed a slight increase (*p* < 0.05) in rumen pH. Dìaz-Carrasco et al. [51], evaluating the effects of chestnuts and quebracho tannins on rumen microbiota, highlighted that tannin addiction increased ruminal pH.

Furthermore, all the extracts depressed methane production related to incubated organic matter and organic matter degradability. This was particularly true of Cs/1/1 and Cs/2/1 supplementation, and appeared to correlate with acetate and propionate production data (Figure 7). Hydrolysable tannins, as well as condensed ones, decreased the total methanogen population, with a positive effect on methane production [16]. Generally, the ability of polyphenols to reduce methane emissions could be due to the decrease in NDF degradation, which leads to a reduction in acetate synthesis, and eventually, to a decrease in the availability of electron donors for the methanogens [52]. Indeed, the reduction in acetate with the increase in propionate plays a key role in establishing an internal hydrogen balance by regulating methanogenesis. The formation of acetate from pyruvate produces metabolic hydrogen, the main substrate of methanogenesis, which is consumed by the formation of propionate from pyruvate [53]. After 24 h of incubation, acetic acid was reduced in all samples tested, with the sole exception of Cs/3/2-50 mg, which favors a 3.7% increase over the control diet. Instead, propionate increased significantly following treatment with Cs/1/1, and decreased only by Cs/3/2-50 mg. The higher molar proportion of propionate in the in vitro fermentation system may be associated with a lower count of protozoa produced by tannins [47,50]. Unsurprisingly, the Cs/3/2-50 mg fraction showed the lowest inhibition of methanogenesis and the highest increase in the A/P ratio, which was reduced by all treatments. Total VFAs increased to the highest percentage with Cs/2/1-200 mg treatment, followed by alcoholic extract at both dosages, which increased by between 32.2% and 30.5%. In contrast, *Castanea sativa* leaf reduced total VFAs with a higher percentage of propionate after 24 h incubation, producing low total gas and CH_4_/digestible organic matter. This, as previously hypothesized, could be achieved without a significant reduction in bacterial and protozoal counts [42]. Unlike the 120 h of incubation, during the first 24 h, butyric acid underwent a notable decrease, mainly at the dose of 200 mg of both the Cs/1/1 extract and its Cs/3/2 fraction. These results are only partially in line with Castro-Montoya et al. [48], who, testing different doses of chestnut tannins (0.5, 0.75, 1 mg/mL) for 24 h of incubation, found a significant decrease in acetate and A/P with a linear increase in propionate. An opposite trend was obtained for total VFAs, butyrate, valerate, and branched-chain fatty acids (BCFA). In addition, valerate increased at all doses tested by up to 50.3% after treatment with Cs/2/1-200 mg.

After 120 h of incubation, while some parameters maintained their trend (e.g., total VFAs, acetate, propionate, iso-valerate), others showed an inversion (e.g., pH, butyrate, valerate, iso-butyrate). Furthermore, the total VFAs increased, and the observed effect for all the tested samples appeared to be dose-dependent (Table 7), except for Cs/3/2. Cs/2/1-200 mg increased the VFA content by a percentage of 54.2% compared to the control diet, followed by Cs/3/2-50 mg, which was able to increase its VFAs by 1.4 times. However, previous reports on chestnut wood tannins (CTWs) highlighted that CTWs did not significantly increase total VFAs [45], or even observed a reduction in VFAs with CTWs [43,54]. The significant effect on VFAs in this study was probably attributable to the high flavonoid content of chestnut leaves [15]. Among the main volatile fatty acids (acetate, propionate, and butyrate), only for propionic and butyric acid was an increase recorded for all the dose fractions tested, while acetate decreased. Propionate increased in various degrees; the Cs/1/1 extract at 50 and 200 mg dose levels was able to increase it by 42.4 and 64.9%, respectively. Both dose levels of Cs/2/1 fraction enhanced propionic acid production, whereas Cs/3/2 resulted in a dose-dependent reduction. Data on acetate levels could be justified by the ability of compounds therein to inhibit acetate-producing bacteria (e.g., *Ruminococcus albus*, *Butyrivibrio fibrisolvens*), either by direct action or by inhibiting the production of their substrates [48]. To support this hypothesis, in a previous study testing increasing levels (0, 5, 10, 15, and 20 g/kg of diet DM) of *A. mearnsii* CTs in the diets of Jersey steers, it was found that the ruminal protozoa population was not affected by CTs, while protein digestibility, ruminal pH, and acetate proportion decreased [55]. The A:P ratio appeared to significantly (*p* < 0.001) decrease at all diet dose levels. Furthermore, Cs/1/1-50 mg and Cs/2/1 at both doses showed a similar increase in butyrate. Moreover, a non-significant variation intra-dose of butyrate was recorded for the Cs/3/2 fraction. The Cs/3/2-200 mg dose level showed the lowest decrease in acetate, with a value equal to 60.3% VFA. It was seen that a chestnut/quebracho tannin supplementation (ratio 1:2) was effective in reducing ruminal NH_3_-N concentration and molar proportion of branched-chain fatty acids, providing a decrease in amino acid deamination [56]. Herein, the same trend was observed for BCFAs, which decreased at all the tested doses (*p* < 0.001), mainly at the Cs/3/2-50 mg dose level. Iso-valerate is a branched-chain VFA from the deamination of leucine, and in the present study, its proportion decreased after only 24 h of incubation at greater doses of polyphenols (fraction Cs/3/2). The decrease in iso-valerate with a higher dose of tannins may indicate a reduction in ruminal protein degradation [54]. Analogously, iso-butyrate, which showed an increase after 24 h, decreased (*p* < 0.05) after 120 h, but not as significantly as iso-valerate. Fraction Cs/2/1 further modified some fermentation parameters (e.g., pH, total VFAs, propionic acid, butyric acid, and valeric acid) for its terpenoid constituents, which are able to influence fermentation process with effects also on the methanogenesis [57] and milk production [58]. Valerate, as opposed to 24 h of incubation, was significantly reduced in all tested fractions, mainly by means of the Cs/3/2-200 mg sample.

The dendrograms related to the results of total VFAs and related to a diet at the dose level of 50 and 200 mg are in Figure S11A. Both dendrograms showed three clusters: the first group included total VFA and acetic acid, the second consisted of propionic and butyric acid, and the third included two subgroups with valeric acid, A/P, BCFA, iso-valeric acid (IIIa) and iso-butyric acid (IIIb). Principal component analysis (PCA) (Figure S11B) at 50 mg showed the positive correlation of total VFA with the enriched polyphenol fraction (Cs/3/2), which showed the highest amount of TFC (*r* = 0.998) and TPC (*r* = 0.913) and acetate at the apolar fraction (named Cs/2/1) (*r* = 0.207 in correlation with TFC; *r* = −0.153 in correlation with TPC). An opposite trend was displayed at the 200 mg dosage. Other volatile fatty acids showed similar distribution at both strengths, with the exception of propionate and butyrate.

## 3. Material and Methods

### 3.1. Plant Collection, Fractionation, and Evaluation of Leaf Chemical Composition

The leaves of *C. sativa* Mill. were collected in August 2021. Voucher specimens were deposited in the private herbarium of CZ (CZ-20190628A-1) and in the Herbarium of Kiel University (KIEL0005015); leaves for phytochemical analyses and twigs for the vouchers were collected in the Botanical Garden of the Pharmaceutical Institute of Kiel University in Kiel/Schleswig-Holstein/Germany, N 54°20′00.8″, E 10°06′58.3″, 33 m a.m.s.l., 28.06.2019, leg.: C. Zidorn, det.: C. Zidorn. The identity of the single cultivated tree was confirmed using the German standard reference flora by Jäger [59]. 

The leaves were first lyophilized and pulverized using a rotating knife homogenizer. Dried leaves underwent ultrasound-assisted maceration (UAM; Branson UltrasonicsTM BransonicTM M3800-E; Danbury, CT, USA) using methanol as extractive solvent. The raw material/solvent ratio was 1:5 (g raw material: mL solvent). Three sonication cycles were carried out, each one of 30 min, to obtain the Cs/1/1 extract. The extract yield (%) was equal to 24.3% (103.7 g). The alcoholic extract was then dissolved in a biphasic solution CHCl_3_:MeOH:H_2_O (13:7:6, *v*:*v*:*v*), and discontinuous liquid–liquid extraction (LLE) was performed. Thus, an organic fraction (Cs/2/1; 16.2% of Cs/1/1) and a hydroalcoholic one (Cs/2/2) were obtained. The fraction Cs/2/2 was further chromatographed on XAD-4 resin, using water first and then methanol. The alcoholic fraction Cs/3/2 was obtained with a 26.6% yield.

Chestnut leaves were also analyzed according to the procedures of the Association of Official Agricultural Chemists [60] to determine dry matter (DM), ether extract (EE), crude protein (CP) and ash. The fiber fractions (neutral detergent fiber on organic matter basis, NDFom; acid detergent fiber on organic matter basis, ADFom; acid detergent lignin, ADL) were also determined according to Van Soest et al. [61].

### 3.2. UHPLC-HRMS and MS/MS Parameters and UV-Vis Analyses

The alcoholic extract, Cs/1/1, and the fractions therefrom were first analyzed by UV-Vis spectrophotometry in the range 200–800 nm by a Cary 100 spectrophotometer. The three samples (10 mg/mL) were profiled by a NEXERA UHPLC system (Shimadzu; Tokyo, Japan) equipped with Luna^®^ Omega C-18 column (1.6-µm particle size, 50 × 2.1 mm i.d.) and 2.0 µL of each sample were injected. The separation was achieved using a binary solution: (A) H_2_O and (B) CH_3_CN both with 0.1% formic acid (HCOOH). A linear gradient was used in which the percentage of solvent B increased as follows: 0–3 min, 2% B; 3–7 min, 2% → 10% B; 7–15 min, 10% → 30% B; 15–20 min, 30% → 45% B; 20–23 min, 45% → 75% B, 23–25 min, 75% → 95% B; 25–27 min, 95% B and 27.01–29.00 min, column re-equilibration. The flow rate was set at 500 µL/min. The AB SCIEX TripleTOF^®^ 4600 (AB Sciex, Concord, ON, Canada) system was equipped with a DuoSprayTM ion source, with the ESI probe used for MS investigations in negative ionization mode, and the APCI probe used for fully automatic mass calibration, using the calibrant delivery system (CDS). A full-scan time-of-flight (TOF) survey (dwell time 250 ms, 100–1500 Da) and eight IDA MS/MS scans (dwell time 100 ms, 80–1300 Da) were acquired, using the following parameters: curtain gas (CUR) 35 psi, nebulizer (GS1) and heated (GS2) gases 60 psi, ion spray voltage (ISVF) 4500 V, ion source temperature (TEM) 600 °C, and declustering potential (DP) −80 V. The collision energy (CE) applied was −45 V, with a collision energy spread (CES) of 15 V. The instrument was controlled by Analyst^®^ TF 1.7 software (AB Sciex, Concord, ON, Canada, 2016), while data processing was carried out using PeakView^®^ software version 2.2 (AB Sciex, Concord, ON, Canada, 2016).

### 3.3. Antioxidant Assessment

The leaf extract and its fractions were tested at 200, 100, 50, 25, 12.5, 6.25, and 3.125 µg/mL (final concentration), by assessing ABTS [2,2′-azinobis-(3-ethylbenzothiazolin-6-sulfonic acid)] radical cation scavenging capacity and 2,2′-diphenyl-1-picrylhydrazyl (DPPH) radical scavenging capability. Ferricyanide PFRAP assay was also carried out. Each test was performed for three replicate measurements. Recorded activities were compared to a blank treated in the same manner as the samples. The ID_50_ and TEAC values were calculated.

#### 3.3.1. Determination of DPPH (2,2′-Diphenyl-1-Picrylhydrazyl) Radical Scavenging Capacity

Samples were mixed into a DPPH^●^ methanol solution (9.4 × 10^−5^ M). Mixtures were stirred for 15 min; after that, the absorption was read at 517 nm by a Wallac Victor3 spectrophotometer with reference to a blank. The results were expressed in terms of the percentage reduction in the initial radical adsorption by the tested samples [62]. Trolox (2, 4, 8, 16, and 32 µM) was used as positive standard.

#### 3.3.2. Determination of ABTS [2,2′-Azinobis-(3-Ethylbenzothiazolin-6-Sulfonic Acid)] Radical Cation Scavenging Capacity

The radical cation ABTS, previously produced by reacting (2,2′-azinobis-(3-ethylbenzothiazolin-6-sulfonic acid); 7 mM) and potassium persulfate (K_2_S_2_O_8_; 2.45 mM), in the dark for 12 h, was diluted with phosphate-buffered saline (pH 7.4) until it reached an absorbance equal to 0.7 at 734 nm. Thus, samples were added to the ABTS^●+^ solution. After 6 min, the absorbance was measured using a Wallac Victor3 spectrophotometer in reference to a blank [62]. Trolox (2, 4, 8, 16, and 32 µM) was used as positive standard.

#### 3.3.3. Determination of Potassium Ferricyanide Reducing Power (PFRAP)

The ability to reduce the Fe(III) of chestnut leaf Cs/1/1 extract and its fractions were estimated using the ferricyanide FRAP assay, according to PFRAP procedure. The absorbance was measured at 700 nm and the increase in absorbance with reference to the blank was considered to value the reducing power [63]. Trolox (2, 4, 8, 16, 32 µM) was used as positive standard.

### 3.4. Determination of Total Phenolic Content

The total phenolic content (TPC) was measured according to the Folin-Ciocalteau procedure [62]. Samples (0.25 and 0.125 mg) were mixed with Na_2_CO_3_ (2.25 mL; 7.5% *w*/*v*) and Folin-Ciocalteu reagent (0.25 mL) and allowed to stand for 3 h at room temperature. The absorbance was read at 765 nm using a Synergy spectrophotometer (Biotek, Winooski, VT, USA). Data were expressed as milligrams of gallic acid equivalents (GAEs) per g of extract. To this purpose, a gallic acid calibration curve (*y* = 0.0247x − 0.0063; *R^2^* = 0.9998) was built up in the range 0.78–25 µg/mL (final concentration levels).

### 3.5. Determination of Total Flavonoid Content

The total flavonoid content (TFC) was determined, with NaNO_2_ (5%, *w*/*v*; 0.3 mL) previously solubilized into 5 mL of distillate water being added to the samples. After 10 min, AlCl_3_ solution (10%, *w*/*v*; 0.6 mL) was added. The reaction was carried out for 6 min. Then, NaOH aqueous solution (1.0 M, 2.0 mL) was added, and the mixture was further diluted to 10 mL with distillate water. The absorbance was read at 510 nm against the blank (water) using a Synergy spectrophotometer (Biotek, Winooski, VT, USA). The flavonoid content was expressed as milligrams of quercetin equivalents (QUEs) per g of extract. To this purpose, a quercetin calibration curve (*y* = 0.0243x − 0.0038; *R^2^* = 0.9978) was built up in the range 0.78–50 µg/mL (final concentration levels).

### 3.6. In Vitro Fermentation

The alcoholic extract of *C. sativa* Mill. (Cs/1/1), fractions Cs/2/1 and Cs/3/2, and a standard diet (as control), were tested to evaluate fermentation characteristics and kinetics by means of the in vitro gas production technique [64]. The extracts were incubated for 24 and 120 h at two dosage levels (50 and 200 mg) with the control diet (1.0055 ± 0.0024 g). This latter consisted in corn silage, oat hay, and concentrate (NDF: 44.2% and CP: 13.7%). All the samples were incubated in hermetically closed serum flasks (120 mL each, three replications for each extract and dosage (3 × 3 × 2)) with rumen fluid (10 mL) at 39°C under anaerobic conditions, and buffered medium (75 mL) and reducing agent (4 mL) were added [65]. The rumen fluid was collected in a pre-warmed thermos at a slaughterhouse, as authorized according to EU legislation [66], from six healthy young bulls (*Bos taurus*) to increase the variability of the microbial population. All procedures concerning animals were accepted by the Ethical Animal Care and Use Committee of the University of Napoli Federico II (Prot. 2019/0013729 of 08/02/2019). Then, the collected rumen liquor was transferred to the laboratory of the Department of Veterinary Medicine and Animal Production. There, it was pooled, flushing with CO_2_, filtered through a cheesecloth, and added to the flasks. After 24 h of incubation, the gas-phase from each flask was sampled (3 mL) in duplicate with a gastight syringe and injected into a gas chromatograph (ThermoQuest 8000top Italia SpA, Rodano, Milan, Italy), equipped with a loop TC detector and a packed column (HaySepQ SUPELCO, 3/16-inch, 80/100 mesh) to detect the methane (CH_4_) production related to incubate organic matter (CH_4_, mL/g iOM) and degraded organic matter (CH_4_ (mL/g dOM). The gas produced during the 120 h of incubation was reordered using a manual pressure transducer (Cole and Palmer Instrument Co, Vernon Hills, IL, USA) and related to incubated OM (OMCV, mL/g). After 120 h of incubation, the fermentation was stopped and the pH of the fermentation liquor was measured using a pH meter (ThermoOrion 720 A+, Fort Collins, CO, USA). Then, the organic matter degradability (OMD, %) at both the incubation times was assessed by the weight differences of the incubated OM and the undegraded filtered (sintered glass crucibles; Schott Duran, Mainz, Germany, porosity # 2) residue burned at 550 °C overnight [67].

### 3.7. Fermentation End-Product Assessment

The fermentation liquor of each bottle after 24 and 120 h of incubation was collected to measure the volatile fatty acids (VFAs) content [68]. The collected fermentation liquors were centrifuged at 12,000× *g* for 10 min at 4 °C (Universal 32R centrifuge, Hettich FurnTech Division DIY, Melle-Neuenkirchen, Germany), and the supernatant (1 mL) was mixed with oxalic acid (1 mL: 0.06 mol). The VFA composition was evaluated by gas chromatography (ThermoQuest 8000top Italia SpA, Rodano, Milan, Italy) equipped with a fused silica capillary column (30 m, 0.25 mm ID, 0.25 μm film thickness) by means of an external standard solution composed of pure acetic, propionic, butyric, iso-butyric, valeric and iso-valeric acids. BCFA percentages were calculated as follows: [(iso-butyric acid + iso-valeric acid)/total VFAs] × 100.

### 3.8. Data Processing and Statistical Analysis

Colorimetric tests were carried out in three replicate measurements for three samples (n = 3) of the extracts (in total, 3 × 3 measurements). All data were expressed as mean ± SD values.

To estimate the fermentation kinetic parameters, the gas production profiles were fitted to the sigmoidal model [69]:

(1) G=A/(1+(Bt)C)

G is the total gas produced (mL/g of incubated OM) at time t (h), A is the asymptotic gas production (mL/g), B is the time at which one-half of A is reached (h), and C is the curve switch. The maximum fermentation rate (R_max_, mL/h) and the time at which it occurred (T_max_, h) were determined using model parameters [70]:

(2) $$R_{\max}=\frac{\left(A\times C^{B}\right)\times \;B\;\times {\;T}_{\max}{}^{\left(B-1\right)}}{{\left(\left(1+C^{B}\right)\times \left(T_{\max}-B\right)\right)}^{2}}$$

(3) Tmax=C×( B−1B+1)1B

Statistical analyses were performed by ANOVA (JMP^®^, Version 14 SW, SAS Institute Inc., Cary, NC, USA, 1989–2019). A post-hoc Dunnett’s test was performed to observe the differences between the control and experimental diets. The significance level was verified at *p* < 0.05, *p* < 0.01, and *p* < 0.001. A statistical comparison Shapiro–Wilk test for normally distributed data was performed. The correlations between the colorimetric assay values and fermentation parameters were also evaluated using the Pearson correlation coefficient (Tables S3 and S4). Dendrograms of fermentation end-product data were made based on mean values of parameters obtained by three fractions (Cs/1/1, Cs/2/1 and Cs/3/2) at both doses (50 and 200 mg). In addition, principal component analyses (PCA), as well as scatter plots, were carried out for two doses by Origin2015 and GraphPad Prism 8.4.2, respectively.

## 4. Conclusions

Chestnut leaf extracts can provide modulation of rumen parameters and can be used as supplements in the livestock diet, thus creating added economic value for the chestnut industry. Chestnut leaves have been shown to be a “reservoir” of structurally different metabolites capable of exerting effects both at 24 and 120 h of incubation, with an increase in total VFAs and propionate and a reduction in BCFA, A/P, acetate, and CH_4_. As for the positive effects on CH_4_ production, one of the most important challenges in livestock is to mitigate the production and emission of this greenhouse gas (GHG). Herein, it has been shown that the alcoholic extract and its polar fraction (Cs/3/2) are rich in polyphenols, including flavonoids and tannins (gallo- and ellagitannins). These are known to be considered “anti-nutritional” because they influence the feed palatability in ruminants by exerting negative effects on rumen fermentation. However, the effects of tannins can be variable depending on the sub-class (condensed or hydrolysable), the plant source and the molecular structure, which plays a key role in determining “structure-effect” in any complex biological system. Furthermore, their effects were also modulated by the synergistic action of flavonoids, which are present in a moderate percentage in the alcoholic extract and Cs/3/2 fraction. However, the non-polar fraction (Cs/2/1) also gave positive results, which were attributable to the presence of fatty acids and triterpenes. Therefore, in the light of the data obtained, it is essential to accurately attribute specific effects to an extract consisting of a well-defined class of metabolites, even better if they are pure molecules. The dose–response efficacy of the chestnut extract/fractions and the evaluation of pure isolated compounds are in line with our future perspectives.

## Figures and Tables

**Table 1:** Quinic acid and phenolic acid glycosides tentatively identified in the chestnut Cs/1/1 alcoholic extract and its Cs/2/1 and Cs/3/2 fractions. RT = retention time; RDB = ring double bond equivalent value. Base peak fragments are reported in bold. ● Detected (Grey color box = not detected).

Peak	Rt	Tentative Assignment	Formula	[M-H]^−^ Found (*m*/*z*)	Error (ppm)	RDB	Cs/1/1	Cs/2/1	Cs/3/2
**1**	0.288	Quinic acid	C_7_H_12_O_6_	191.0562	0.5	2	●		●
**10**	1.260	Dihydroxybenzoic acid hexoside	C_13_H_16_O_9_	315.0724	0.8	6	●		●
**20**	3.929	*p*-Coumaric acid hexoside I	C_15_H_18_O_8_	325.0926	−0.9	7			●
**22**	4.609	*p*-Coumaric acid hexoside II	C_15_H_18_O_8_	325.0926	−0.9	7	●		●
**24**	4.988	Caffeic acid hexoside	C_15_H_18_O_9_	341.0871	−1.2	7			●
**26**	5.628	5-*O*-Caffeoyl quinic acid	C_16_H_18_O_9_	353.0884	1.7	8	●		●
**35**	7.080	3-*O*-*p*-Coumaroyl quinic acid	C_16_H_18_O_8_	337.0925	−1.2	8	●		●
**36**	7.218	3-*O*-*p*-Coumaroyl quinic acid	C_16_H_18_O_8_	337.0933	1.2	8	●		●
**56**	10.379	Benzyl-dihydroxybenzoate-*O*-pentosyl hexoside I	C_25_H_30_O_13_	537.1624	1.9	11	●		●
**59**	10.635	Benzyl-dihydroxybenzoate-*O*-hexoside	C_20_H_22_O_9_	405.1192	0.2	10	●	●	●
**74**	13.160	Benzyl-dihydroxybenzoate-*O*-pentosyl hexoside II	C_25_H_30_O_13_	537.1635	4.0	11	●	●	●

**Table 2:** Gallo- and ellagitannin compounds (and their metabolic precursors) tentatively identified in the chestnut Cs/1/1 alcoholic extract and its fractions. Rt = retention time. Base peak fragments are reported in bold. ● Detected (Grey color box = not detected).

Peak	Rt	Tentative Assignment	Formula	[M-H]^−^ Found (*m*/*z*)	Error (ppm)	Cs/1/1	Cs/2/1	Cs/3/2
**2**	0.602	Galloyl hexose I	C_13_H_16_O_10_	331.0670	−0.2	●		●
**3**	0.680	Pyrogallol gallic acid	C_13_H_10_O_8_	293.0302	0.1	●		●
**4**	0.680	Hexahydroxydiphenic acid	C_14_H_10_O_10_	337.0204	0.8	●		●
**5**	0.721	Gallic acid	C_7_H_6_O_5_	169.0151	5.0			●
**6**	0.776	Galloyl hexose II	C_13_H_16_O_10_	331.0669	−0.5			●
**7**	1.008	Digalloyl hexose I	C_20_H_20_O_14_	483.0785	1.0			●
**8**	1.124	3,4,5-trihydroxybenzyl hexoside I (e.g., crenatin)	C_13_H_18_O_9_	317.0879	0.3	●		●
**9**	1.163	NHTP-HHDP-hexose I	C_41_H_26_O_26_	933.0640; 466.0328 [M-2H]^2−^	1.8	●		●
**11**	1.417	3,4,5-trihydroxybenzyl hexoside II	C_13_H_18_O_9_	317.0877	−0.3			●
**12**	1.535	Galloyl hexose III	C_13_H_16_O_10_	331.0674	1.0			●
**13**	1.632	Bis-HHDP-hexose I	C_34_H_24_O_22_	783.0700	1.7	●		●
**14**	1.768	Galloyl shikimic acid I	C_14_H_14_O_9_	325.0554	−3.4			●
**15**	1.827	NHTP-HHDP-hexose II	C_41_H_26_O_26_	933.0641; 466.0326 [M-2H]^2−^	0.2	●		●
**16**	2.087	Galloyl shikimic acid II	C_14_H_14_O_9_	325.0563	−0.6			●
**17**	2.187	Galloyl dihydroxybenzoic acid	C_14_H_10_O_8_	305.0305	0.7			●
**18**	2.385	Galloyl shikimic acid III	C_14_H_14_O_9_	325.0566	−0.1			●
**19**	3.314	Bis-HHDP-hexose II	C_34_H_24_O_22_	783.0696	1.2			●
**21**	4.589	Galloyl-chebuloyl-HHDP-hexose I	C_41_H_30_O_27_	953.0901	−0.1			●
**23**	4.966	HHDP-valoneoyl-hexose	C_41_H_28_O_27_	951.0761	1.7	●		●
**25**	5.268	Digalloyl-HHDP-hexose I	C_34_H_26_O_22_	785.0857	1.8	●		●
**27**	6.004	Galloyl-HHDP-hexose	C_27_H_22_O_18_	633.0742	1.8	●		●
**28**	6.004	Galloyl-bis-HHDP-hexose I	C_41_H_28_O_26_	935.0818	2.1			●
**29**	6.244	Methylvaloneoyl–NHTP–hexose	C_49_H_32_O_31_	557.0405 [M-2H]^2−^	1.8	●		●
**30**	6.165	Digalloyl hexose II	C_20_H_20_O_14_	483.0788	1.6	●		●
**31**	6.560	Galloyl-bis-HHDP-hexose II (e.g., stachyurin/casuarinin)	C_41_H_28_O_26_	935.0801	0.5	●		●
**32**	6.500	Galloyl-chebuloyl-HHDP-hexose II (e.g., chebulagic acid)	C_41_H_30_O_27_	953.0909	0.8	●		●
**33**	6.546	Trigalloyl hexose I	C_27_H_24_O_18_	635.0892	0.3	●		●
**34**	6.819	Chesnatin	C_27_H_26_O_18_	637.1064	2.8	●		●
**37**	7.297	Digalloyl-HHDP-hexose II	C_34_H_26_O_22_	785.0856	1.7	●		●
**38**	7.417	Digalloyl deoxyhexose	C_20_H_20_O_13_	467.0843	2.5	●		●
**39**	8.110	Isochesnatin	C_27_H_26_O_18_	637.1063	2.6	●		●
**40**	8.327	Galloyl phenol hexoside I (e.g., cretanin)	C_20_H_22_O_13_	469.0996	1.8	●		●
**41**	8.581	Galloyl-bis-HHDP-hexose III	C_41_H_28_O_26_	935.0797; 467.0375 [M-2H]^2−^	0.1	●		●
**42**	8.721	Galloyl phenol hexoside II	C_20_H_22_O_13_	469.0997	2.0	●		●
**43**	8.919	Trigalloyl hexose II	C_27_H_24_O_18_	635.0905	2.4	●		●
**44**	9.000	Trigalloyl-valoneoyl-HHDP-hexose	C_48_H_34_O_31_	552.0490 [M-2H]^2−^	1.1	●		●
**45**	9.455	Trigalloyl-HHDP-hexose	C_41_H_30_O_26_	937.0947; 468.0454 [M-2H]^2−^	−0.6	●		●
**47**	9.704	Tetragalloyl hexose	C_34_H_28_O_22_	787.1007	1.0	●		●
**48**	9.704	Ellagic acid pentoside	C_19_H_14_O_12_	433.0415	0.6	●		●
**49**	9.754	Chestanin	C_40_H_42_O_26_	937.1894	0.3	●		●
**58**	10.592	Isochestanin	C_40_H_42_O_26_	937.1904	1.3	●		●
**69**	11.980	Trimethylellagic acid hexose	C_23_H_24_O_13_	551.057 [M+FA]^−^	n.c.	●	●	●
**81**	14.174	Trimethylellagic acid deoxyhexose	C_23_H_22_O_12_	535.1106 [M+FA]^−^	n.c.	●	●	●
**82**	14.174	Trimethylellagic acid I	C_17_H_12_O_8_	343.0455	−1.3	●	●	
**87**	15.139	Trimethylellagic acid II	C_17_H_12_O_8_	343.0459	−0.1	●	●	

**Table 3:** Flavonoid compounds tentatively identified in the chestnut Cs/1/1 alcoholic extract and its Cs/2/1 and Cs/3/2 fractions. Rt = retention time. Base peak fragments are reported in bold. ● Detected (Grey color box = not detected).

Peak	Rt	Tentative Assignment	Formula	[M-H]^−^ Found (*m*/*z*)	Error (ppm)	Cs/1/1	Cs/2/1	Cs/3/2
**46**	9.595	Quercetin pentosyl-hexoside	C_26_H_28_O_16_	595.1318	2.3	●		●
**50**	9.969	Quercetin hexuronide I	C_21_H_18_O_13_	477.0686	2.6	●		●
**51**	9.911	Quercetin 3-*O*-hexoside I	C_12_H_20_O_12_	463.0893	2.4	●		●
**52**	10.009	Rutin	C_27_H_30_O_16_	609.1479	2.9	●		●
**53**	10.067	Quercetin 3-*O*-hexoside II	C_12_H_20_O_12_	463.0892	2.2	●		●
**54**	10.315	Quercetin hexuronide II	C_21_H_18_O_13_	477.0680	1.1	●		●
**55**	10.315	Quercetin 3-*O*-hexoside III	C_12_H_20_O_12_	463.0890	1.7	●		●
**57**	10.515	Quercetin 3-*O*-pentoside	C_20_H_18_O_11_	433.0786	1.5	●		●
**60**	10.810	Kaempferol deoxyhexosyl-hexoside I	C_27_H_30_O_15_	593.1533	3.5	●		●
**61**	10.887	Isorhamnetin deoxyhexoside I	C_21_H_20_O_11_	447.0941	1.8	●		●
**62**	11.099	Isorhamnetin deoxyhexosyl-hexoside I	C_28_H_32_O_16_	623.1632	2.2	●		●
**63**	11.176	Kaempferol deoxyhexosyl-hexoside II	C_27_H_30_O_15_	593.1526	2.4	●		●
**64**	11.196	Isorhamnetin 3-*O*-hexoside I	C_22_H_22_O_12_	477.1046	1.6	●		●
**65**	11.293	Isorhamnetin deoxyhexoside II	C_21_H_20_O_11_	447.0935	0.5	●		●
**66**	11.293	Isorhamnetin hexuronide	C_22_H_20_O_13_	491.0841	2.0	●		●
**67**	11.510	Isorhamnetin deoxyhexosyl-hexoside II	C_28_H_32_O_16_	623.1633	2.3	●		●
**68**	11.570	Isorhamnetin 3-*O*-hexoside II	C_22_H_22_O_12_	477.1051	2.6	●		●
**70**	12.181	Kaempferol acetylhexoside I	C_23_H_22_O_12_	489.1058	4.0	●	●	●
**71**	12.402	Quercetin	C_15_H_10_O_7_	301.0354	−0.3	●	●	●
**72**	12.737	Luteolin	C_15_H_10_O_6_	285.0398	−2.3	●	●	●
**73**	12.819	Kaempferol acetylhexoside II	C_23_H_22_O_12_	489.1051	2.6	●		●
**75**	13.729	Quercetin *p*-coumaroyl-hexoside	C_30_H_26_O_14_	609.1258	1.3	●		●
**76**	13.749	Quercetin *p*-coumaroyl- deoxyhexosyl-hexoside I	C_36_H_36_O_18_	755.1854	3.3	●		●
**77**	13.769	Kaempferol *p*-coumaroyl-hexoside I	C_30_H_26_O_13_	593.1326	2.4	●	●	●
**78**	13.968	Quercetin hexuronide butyl-ester	C_25_H_26_O_14_	533.1316	2.9			●
**79**	13.968	Quercetin *p*-coumaroyl- deoxyhexosil-hexoside II	C_36_H_36_O_18_	755.1850	3.3	●		●
**80**	13.984	Kaempferol	C_15_H_10_O_6_	285.0403	−0.6	●	●	●
**83**	14.366	Kaempferol *p*-coumaroyl deoxyhexosyl hexoside I	C_36_H_36_O_17_	739.1894	1.9	●		●
**85**	14.561	Kaempferol *p*-coumaroyl-hexoside II	C_30_H_26_O_13_	593.1311	1.7	●	●	●
**86**	14.643	Kaempferol *p*-coumaroyl deoxyhexosyl hexoside II	C_36_H_36_O_17_	739.1899	2.9	●		●
**88**	15.563	Kaempferol acetyl *p*-coumaroyl-hexoside	C_32_H_28_O_14_	635.1432	4.0	●	●	●
**89**	15.799	Methylkaempferol	C_16_H_12_O_6_	299.0559	−0.7	●	●	●
**91**	17.088	Kaempferol di-*p*-coumaroyl hexoside I	C_39_H_32_O_15_	739.1695	3.6	●	●	●
**92**	17.284	Kaempferol di-*p*-coumaroyl hexoside II	C_39_H_32_O_15_	739.1694	3.3	●	●	●
**93**	17.313	Acacetin	C_16_H_12_O_5_	283.0611	−0.3	●	●	
**96**	18.337	Kaempferol acetyl di-*p*-coumaroyl hexoside I	C_41_H_34_O_16_	781.1792	2.3	●	●	●
**97**	18.594	Kaempferol acetyl di-*p*-coumaroyl hexoside II	C_41_H_34_O_16_	781.1797	2.9	●	●	●
**100**	19.954	Kaempferol di-acetyl di-*p*-coumaroyl hexoside I	C_43_H_36_O_17_	823.1879	−0.1	●	●	
**102**	20.144	Kaempferol di-acetyl di-*p*-coumaroyl hexoside II	C_43_H_36_O_17_	823.1885	0.6	●	●	

**Table 4:** Lipid compounds tentatively identified in the chestnut Cs/1/1 alcoholic extract and its Cs/2/1 and Cs/3/2 fractions. Rt = retention time; RDB = ring double bond equivalent value. Base peak fragments are reported in bold. ● Detected (Gray color box = not detected).

Peak	Rt	Tentative Assignment	Formula	[M-H]^−^ Found (*m*/*z*)	Error (ppm)	Cs/1/1	Cs/2/1	Cs/3/2
**84**	14.436	Traumatic acid	C_12_H_20_O_4_	227.1291	1.0	●	●	●
**90**	16.019	3,7,24-trihydroxy-cycloartene-28-oic acid hexoside	C_36_H_58_O_10_	695.4037 [M+FA]^−^	n.c.	●	●	●
**94**	17.343	3,7-dihydroxynorcycloartane 24,28-dioic acid (e.g., castaartancrenoic acid A)	C_27_H_42_O_6_	461.2921	2.7	●	●	●
**95**	17.856	Cycloartane-type triterpene	C_30_H_50_O_6_	505.3544	1.9	●	●	●
**98**	18.752	Cycloartane-type triterpene	C_27_H_42_O_5_	445.2956	−0.8	●	●	●
**99**	19.845	Cycloartane-type triterpene	C_30_H_48_O_5_	487.3435	1.2		●	
**101**	20.044	Cycloartane-type triterpene	C_30_H_48_O_5_	487.3437	1.6		●	
**103**	20.256	Cycloartane-type triterpene	C_30_H_48_O_5_	487.3438	1.8		●	
**104**	20.425	Pentacyclic triterpene	C_30_H_48_O_6_	503.3383	1.0		●	
**105**	20.606	DGMG (18:3)	C_33_H_56_O_14_	675.3601	0.5	●	●	●
**106**	20.721	9-oxooctadeca-10,12-dienoic acid	C_18_H_32_O_3_	293.2123	0.3		●	
**107**	20.721	Pentacyclic triterpene	C_30_H_48_O_6_	503.3385	1.4		●	
**108**	21.100	9-oxooctadeca-10,12,15 trienoic acid	C_18_H_28_O_3_	291.1965	−0.2		●	
**109**	21.318	l-PA (18:3)	C_21_H_37_O_7_P	431.2212	1.8	●	●	●
**110**	21.846	Pentacyclic triterpene	C_30_H_48_O_4_	471.3501 517.3558 [M+FA]^−^	4.5	●	●	
**111**	22.313	l-PA (16:0)	C_19_H_39_O_7_P	409.2358	−0.6	●	●	●
**112**	22.513	Pentacyclic triterpene	C_30_H_48_O_4_	471.3487	1.5		●	
**113**	22.654	Pentacyclic triterpene	C_30_H_48_O_5_	487.3440	2.9		●	
**114**	22.695	Pentacyclic triterpene	C_30_H_48_O_4_	471.3480	0.1		●	
**115**	22.784	Pentacyclic triterpene	C_30_H_48_O_5_	487.3433	0.8		●	
**116**	22.975	Linolenic acid	C_18_H_30_O_2_	277.2172	−0.4		●	
**117**	23.115	Pentacyclic triterpene	C_30_H_48_O_4_	471.3483	0.6		●	
**118**	23.215	Pentacyclic triterpene (e.g., ursolic or oleanolic acid)	C_30_H_48_O_3_	455.3537	1.4		●	
**119**	23.496	Linoleic acid	C_18_H_32_O_2_	279.2324	−2.0		●	

**Table 5:** (**A**) In vitro cumulative gas production, organic matter degradability, and fermentation kinetics parameters of different *C. sativa* extracts. OMD: organic matter degradability; OMCV: cumulative volume of gas related to incubated OM. R_max_: maximum fermentation rate; T_max_: time at which R_max_ occurs. * *p* < 0.05, ** *p* < 0.01 and *** *p* < 0.001. ^NS^: not significant. (**B**) In vitro gas production over time of *C. sativa* Cs/1/1 extract and its fractions Cs/2/1 and Cs/3/2 at 50-mg (●) and 200-mg (●) dose levels and control diet (●).

(A)				(B)
	**control diet**	**Cs/1/1**		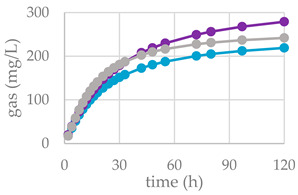
**Parameter**		*50* mg	*200* mg	
**OMD (%)**	74.9	65.0 **	68.9 **	
**OMCV (mL/g)**	244	208 ***	234 ^NS^	
**T_max_ (h)**	3.11	1.49 **	1.24 **	
**R_max_ (mL/h)**	10.6	9.14 ^NS^	10.1 ^NS^	
	**control diet**	**Cs/2/1**		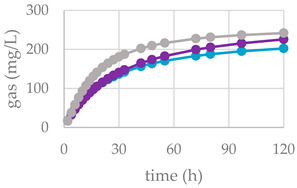
		*50* mg	*200* mg	
**OMD (%)**	74.9	64.9 **	65.9 ***	
**OMCV (mL/g)**	244	194 ***	184 ***	
**T_max_ (h)**	3.11	0.46 ***	0.33 ***	
**R_max_ (mL/h)**	10.6	9.25 ^NS^	8.69 *	
	**control diet**	**Cs/3/2**		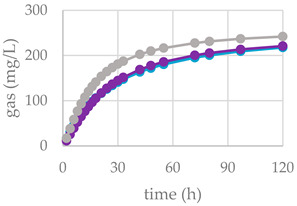
		*50* mg	*200* mg	
**OMD (%)**	74.9	67.7 **	67.5 **	
**OMCV (mL/g)**	244	215 **	219 **	
**T_max_ (h)**	3.11	1.07 **	3.91 ^NS^	
**R_max_ (mL/h)**	10.6	8.17 *	7.04 **	

**Table 6:** In vitro gas production and fermentation rate of different *C. sativa* extracts after 24 h of incubation. OMD: organic matter degradability; CH_4_ (mL/g iOM): methane production related to incubate organic matter; CH_4_ (mL/d OM): mL of methane production related to degraded OM. Along the row ** *p* < 0.01 and *** *p* < 0.001; ^NS^: not significant; MSE: mean square error.

	Control Diet	Cs/1/1		Cs/2/1		Cs/3/2		MSE
		50 mg	200 mg	50 mg	200 mg	50 mg	200 mg	
**OMD**	44.7	44.8 ^NS^	56.3 ***	47.3 ^NS^	54.6 ***	47.0 ^NS^	56.3 ***	0.61
**CH_4_ (mL/g iOM)**	10.3	2.41 ***	2.14 ***	2.56 ***	2.43 ***	6.96 **	6.63 ***	0.15
**CH_4_(mL/d OM)**	23.1	5.38 ***	3.79 ***	5.41 ***	4.45 ***	14.8 **	11.8 ***	0.71

**Table 7:** Effects of *C. sativa* Cs/1/1 extract and its fractions Cs/2/1 and Cs/3/2 at 50-mg and 200-mg dose levels on fermentation end products after 120 h of incubation. Total VFA: total volatile fatty acids (acetate + propionate + butyrate + iso-butyrate + valerate + iso-valerate); AcA = acetic acid; PrA = propionic acid; ButA = Butyric acid; ValA = valeric acid; iso-ButA = iso-butyric acid; iso-ValA = iso-valeric acid; BCFA= branched chain fatty acids (iso-butyrate + iso-valerate/total VFAs); A/P = acetate/propionate. Along the row * *p* < 0.05, ** *p* < 0.01 and *** *p* < 0.001; NS: not significant; MSE: mean square error. In the lower panel, the percentage increase or decrease in each volatile fatty acid is plotted for different tested dose levels (● 50 mg and ● 200 mg) vs. FA% in the control diet.

	Control Diet	Cs/1/1		Cs/2/1		Cs/3/2		MSE
		50 mg	200 mg	50 mg	200 mg	50 mg	200 mg	
pH	6.20	6.46 ***	6.43 ***	6.49 ***	6.35 **	6.34 **	6.31 *	0.0006
Total VFA (mmol/L)	57.2	75.0 ***	76.9 ***	68.7 **	88.2 ***	80.4 ***	73.5 ***	1.33
AcA (% VFA)	64.7	56.8 **	54.8 **	55.5 **	55.9 **	55.8 **	60.3 *	1.59
PrA (% VFA)	15.1	21.5 **	24.9 ***	22.3 ***	22.3 ***	23.5 **	19.7 ***	0.98
ButA (% VFA)	11.9	16.5 *	15.4 *	16.5 *	16.6 *	16.1 *	16.0 *	1.03
ValA (% VFA)	3.98	2.21 ***	2.25 ***	3.05 **	2.28 ***	2.53 **	1.73 ***	0.020
iso-ButA (% VFA)	0.99	0.80 *	0.69 *	0.72 *	0.93 ^NS^	0.77 *	0.62 **	0.002
iso-ValA (% VFA)	3.25	2.17 ***	2.24 ***	1.89 ***	1.96 ***	1.29 ***	1.65 ***	0.003
BCFA (% VFA)	7.42	3.95 ***	3.80 ***	3.08 ***	3.28 ***	2.56 ***	3.09 ***	0.014
A/P	4.27	2.64 ***	2.20 ***	2.50 ***	2.50 ***	2.38 ***	3.06 ***	0.03
	*AcA* *PrA* *ButA* *ValA* *iso-ButA* *iso-ValA*	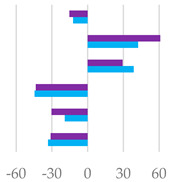		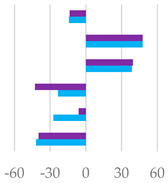		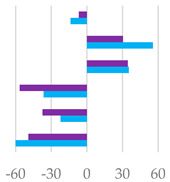		

## Data Availability

Data are within the manuscript and related Supplement Materials.

## Supplementary Materials

The following are available online at https://www.mdpi.com/article/10.3390/molecules27248662/s1. Figure S1: Worldwide distribution of *Castanea sativa* Mill. Figure S2: UV/Visible spectra of fractions Cs/2/1 and Cs/3/2. Figure S3: (A) TOF-MS/MS spectrum of compound **10**; (B) putative fragmentation patterns; theoretical mass is reported under each structure. Figure S4: TOF-MS/MS spectra of (A) compound **4** and (B) compound **3**. Putative fragmentation patterns are depicted; theoretical mass is reported under each structure. Figure S5: Galloyl hexose isomers (**2**, **6**, **12**) TOF-MS/MS spectra. (A) Compound **2**; (B) compound **6**; (C) compound **12**. Putative fragmentation patterns are depicted; theoretical mass is reported under each structure. Figure S6: Digalloyl hexoses (A) **7**, and (B) **30** TOF-MS/MS spectra and their putative fragmentation patterns; theoretical mass is reported under each structure. Figure S7: TOF-MS/MS spectra of compound **17**, tentatively identified as galloyl dihydroxybenzoic acid. Figure S8: TOF-MS/MS spectra of compounds **8** (A) and 11 (B). In panel (C) the putative fragmentation pattern of compound **8** is depicted. Figure S9: TOF-MS/MS spectra of (A) chestanin (**49**) and (B) isochestanin (**58**). Putative fragmentation patterns are depicted; theoretical mass is reported under each structure. Figure S10: TOF-MS/MS spectra of compound **23** with [M-H]^−^ ion at *m*/*z* 951.0907. Structures of observed neutral losses are depicted. Figure S11: (A) Dendrograms of different volatile fatty acids obtained by treatment at 50 (*i.*) and 200 mg (*ii.*); (B) (*i.*) PCA (% variance on PC1 99.8; on PC2 0.2) of VFAs at 50 mg-dose level; (*ii*.) PCA (% variance on PC1 99.6; on PC2 0.4) of VFAs 200 of Cs/1/1, Cs/2/1 and Cs/3/2 fractions. Table S1: Compounds tentatively identified in the chestnut Cs/1/1 alcoholic extract and its Cs/2/1 and Cs/3/2 fractions. Rt = retention time; RDB = ring double bond equivalent value. Base peak fragments are reported in bold. Table S2: Effects of *C. sativa* L. extracts at different doses (50 and 200 mg) on fermentation end products after 24 h of incubation. Total VFA: total volatile fatty acids (acetate + propionate + butyrate + iso-butyrate + valerate + iso-valerate); AcA = acetic acid; PrA = propionic acid; ButA = butyric acid; ValA = valeric acid; iso-ButA = iso-butyric acid; iso-ValA = iso-valeric acid; BCFA= branched chain fatty acids (iso-butyrate + iso-valerate/tVFA); A/P = acetate/propionate. Along the row * *p* < 0.05, ** *p* < 0.01 and *** *p*< 0.001; NS: not significant; MSE: mean square error. Table S3: Values of Pearson coefficient correlation between antiradical (DPPH^●^, ABTS^●+^) activities, reducing activity (PFRAP), total phenol content (TPC), and total flavonoid content (TFC), with fermentation parameters at the dose level of 50 mg. tVFA: total volatile fatty acids; AcA = acetic acid; PrA = propionic acid; ButA = butyric acid; ValA = valeric acid; iso-ButA = iso-butyric acid; iso-ValA = iso-valeric acid; BCFA: branched chain fatty acids (iso-butyrate + iso-valerate/total VFAs); A/P = acetate/propionate; OMD: organic matter degradability; OMCV: cumulative volume of gas related to incubated organic matter. R_max_: maximum fermentation rate; T_max_: time at which R_max_ occurs. Table S4: Values of Pearson’s coefficient correlation, between antiradical (DPPH^●^, ABTS^●+^) activities, reducing activity (PFRAP), total phenol content (TPC), total flavonoid content (TFC) with fermentation parameters at the dose level of 200 mg. tVFA: total volatile fatty acids; AcA = acetic acid; PrA = propionic acid; ButA = butyric acid; ValA = valeric acid; iso-ButA = iso-butyric acid; iso-ValA = iso-valeric acid; BCFA: branched chain fatty acids (iso-butyrate + iso-valerate/total VFAs); A/P = acetate/propionate; OMD: organic matter degradability; OMCV: cumulative volume of gas related to incubated organic matter. R_max_: maximum fermentation rate; T_max_: time at which R_max_ occurs.

## Sample Availability

Samples of the investigated extract and its fractions are available from the authors.
