# Supplementary material for: Castanea sativa Mill. Leaf: UHPLC-HR MS/MS Analysis and Effects on In Vitro Rumen Fermentation and Methanogenesis
Source: Molecules. 2022 Dec 7;27(24):8662. doi: 10.3390/molecules27248662 (PMC9785889; doi:10.3390/molecules27248662)
Supplement: Supplementary file 1 [file molecules-27-08662-s001.zip › molecules-2017641-supplementary.pdf]

Castanea sativa Mill. leaf: UHPLC-ESI-Q*q*TOF analysis and effects on *in vitro* rumen fermentation and methanogenesis

M. Formato,[1] A. Vastolo,[2] S. Piccolella,[1] S. Calabrò,[2] M.I. Cutrignelli,[2] C. Zidorn,[3] S. Pacifico[1]

[1]Department of Environmental, Biological and Pharmaceutical Sciences and Technologies, University of Campania 'Luigi Vanvitelli', Via Vivaldi 43, 81100 Caserta, Italy
[2]Department of Veterinary Medicine and Animal Production, University of Naples Federico II, Via Federico Delpino 1 – 80137, Napoli, Italy
[3]Pharmazeutisches Institut, Abteilung Pharmazeutische Biologie, Christian-Albrechts-Universität zu Kiel, Gutenbergstraße 76, 24118 Kiel, Germany

Supplement Materials

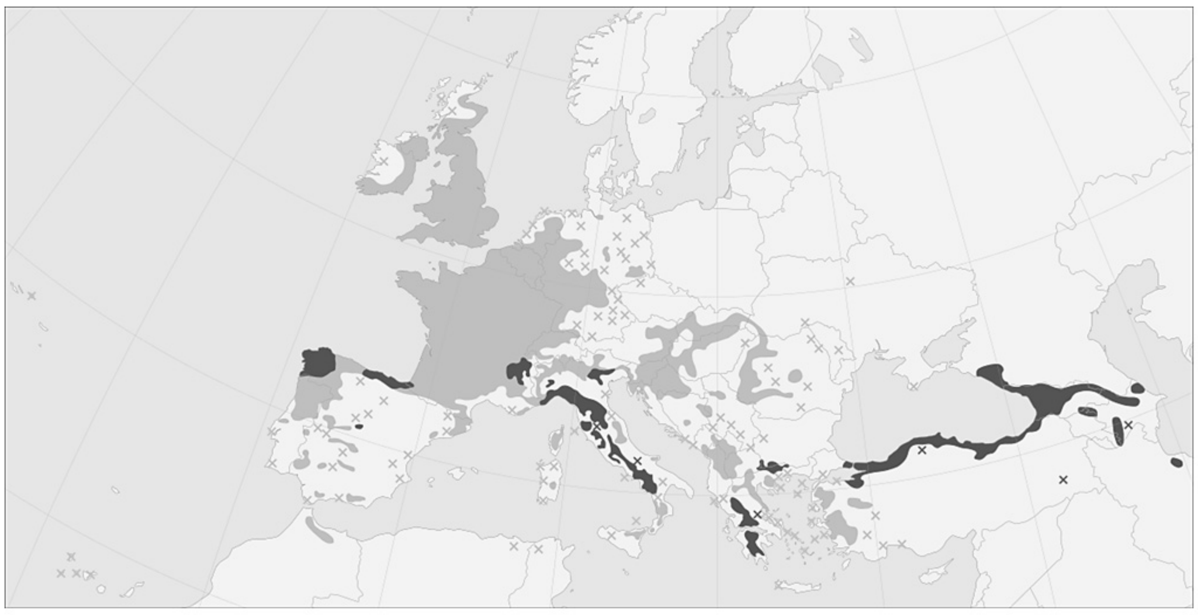

Probable native range and isolated population.

Introduced and naturalized (synanthropic) area and isolated population since Neolithic.

Figure S1. Worldwide distribution of Castanea sativa Mill. (readapted from https://www.euforgen.org/species/castanea-sativa/)

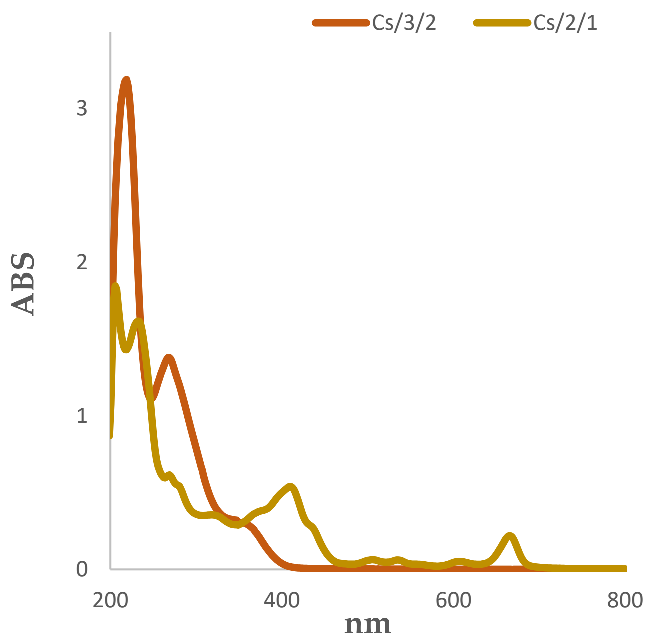

Figure S2. UV/Visible spectra of fractions Cs/2/1 and Cs/3/2.

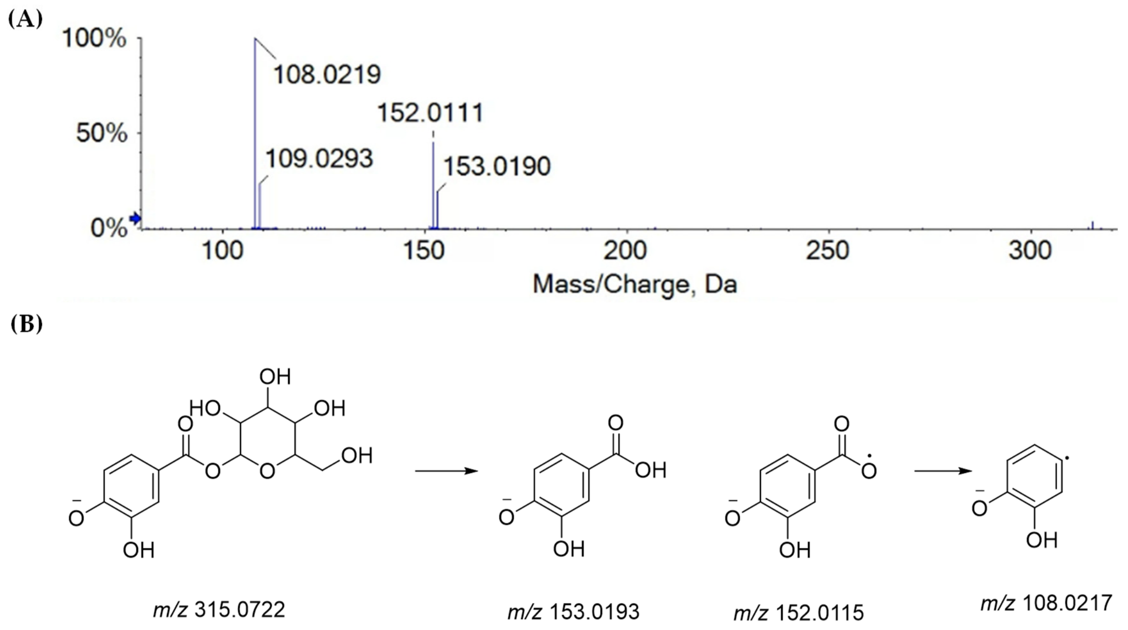

Figure S3. (A) TOF-MS/MS spectrum of compound **10**, **(B)** putative fragmentation patterns; theoretical mass is reported under each structure.

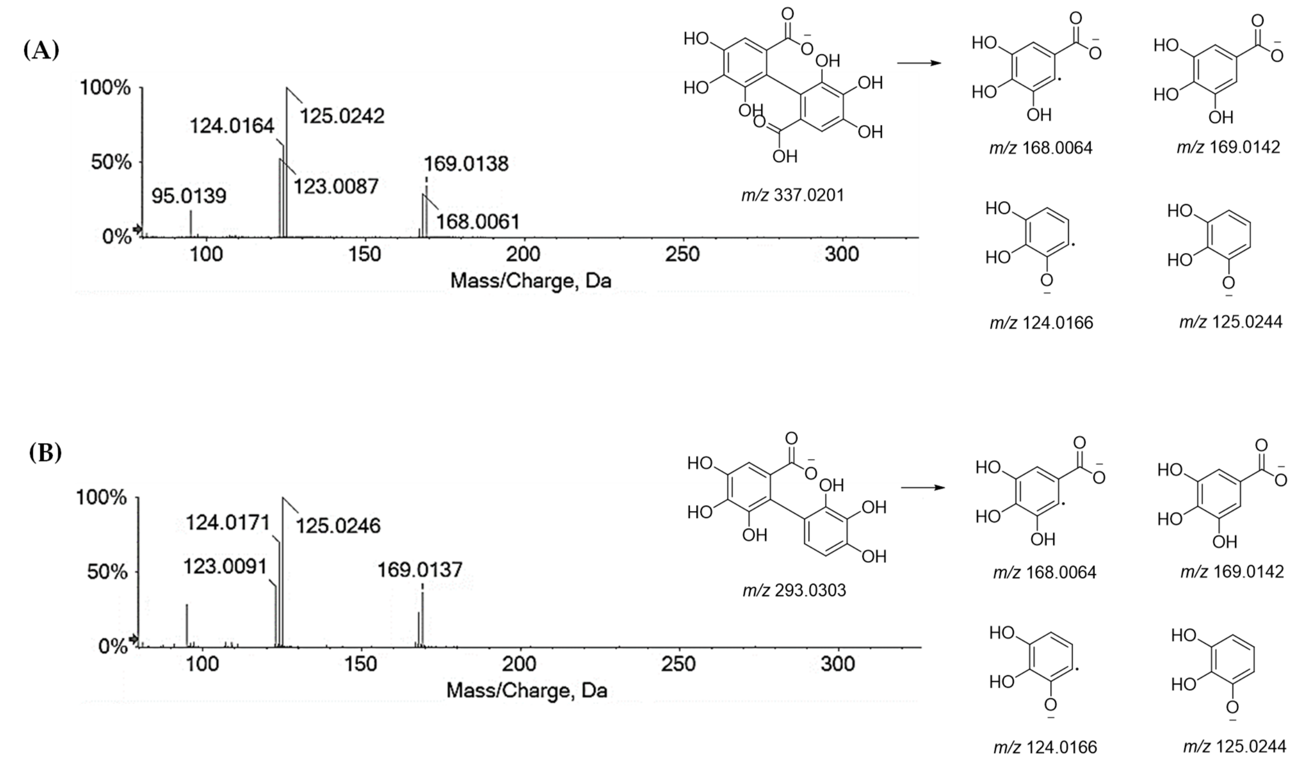

Figure S4. TOF-MS/MS spectra of **(A)** compound **4** and **(B)** compound **3.** Putative fragmentation patterns are depicted; theoretical mass is reported under each structure.

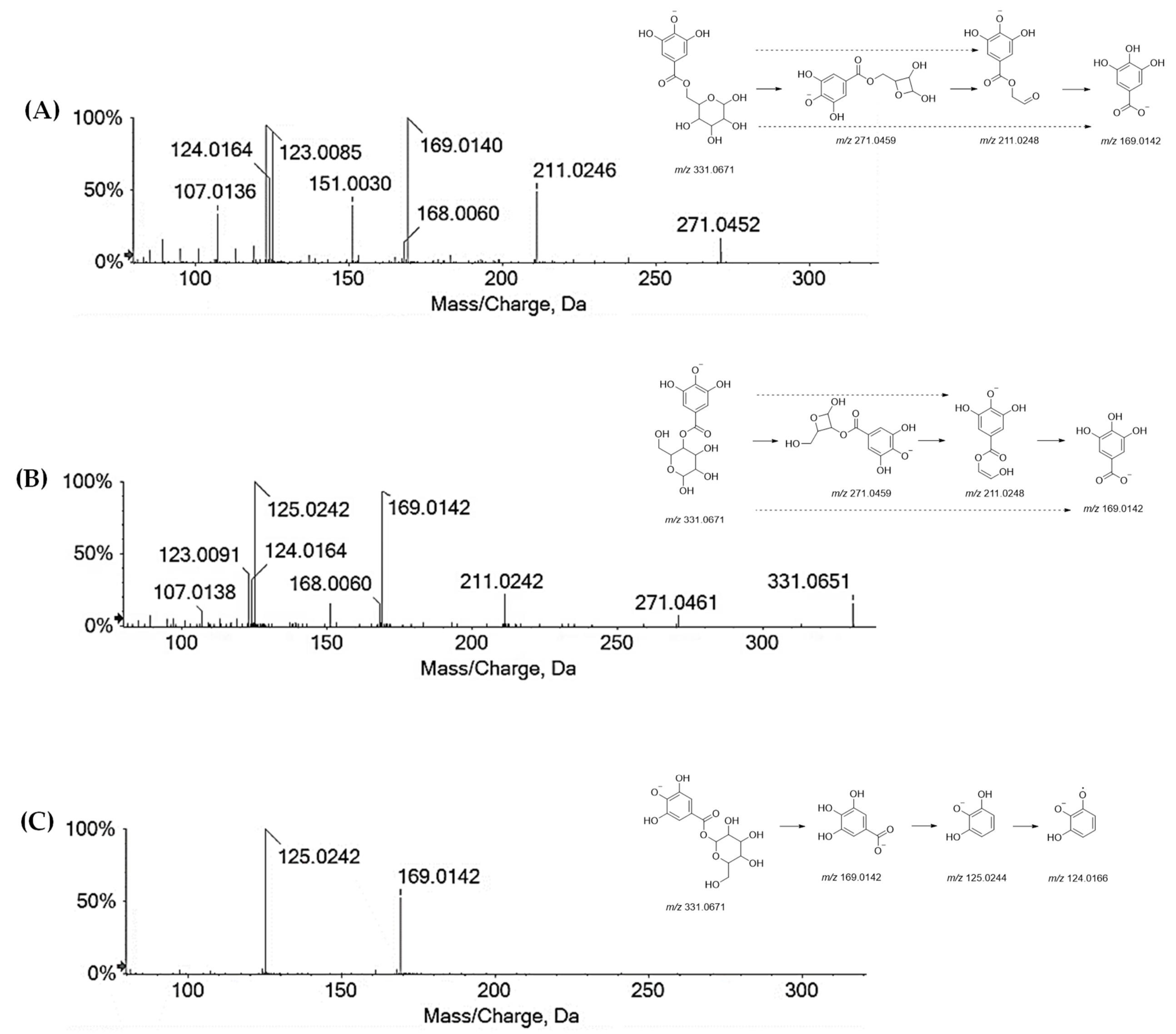

Figure S5. Galloyl hexose isomers (**2, 6, 12**) TOF-MS/MS spectra. **(A)** Compound **2**; **(B)** compound **6**; **(C)** compound **12**. Putative fragmentation patterns are depicted; theoretical mass is reported under each structure.

(A)

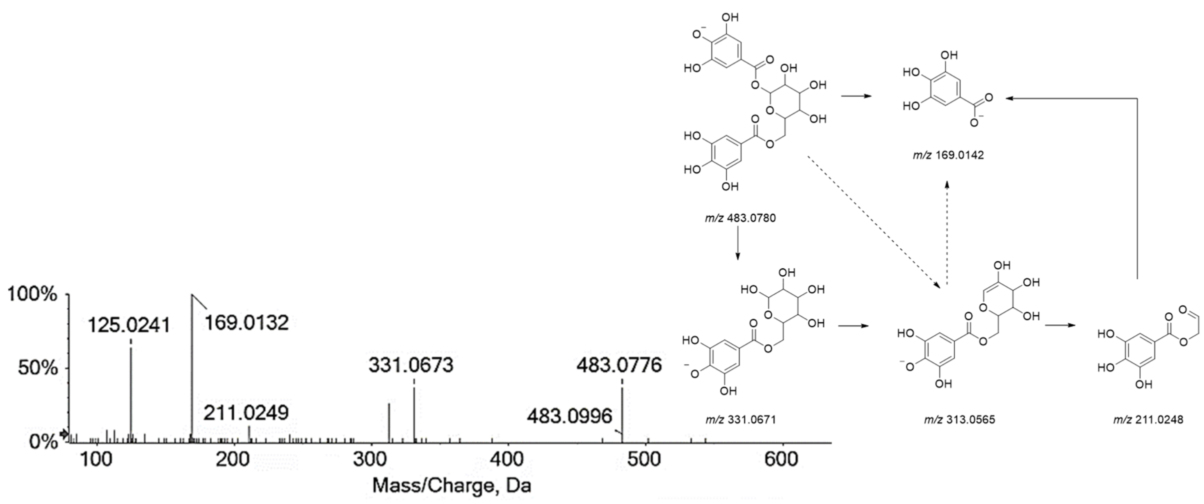

(B)

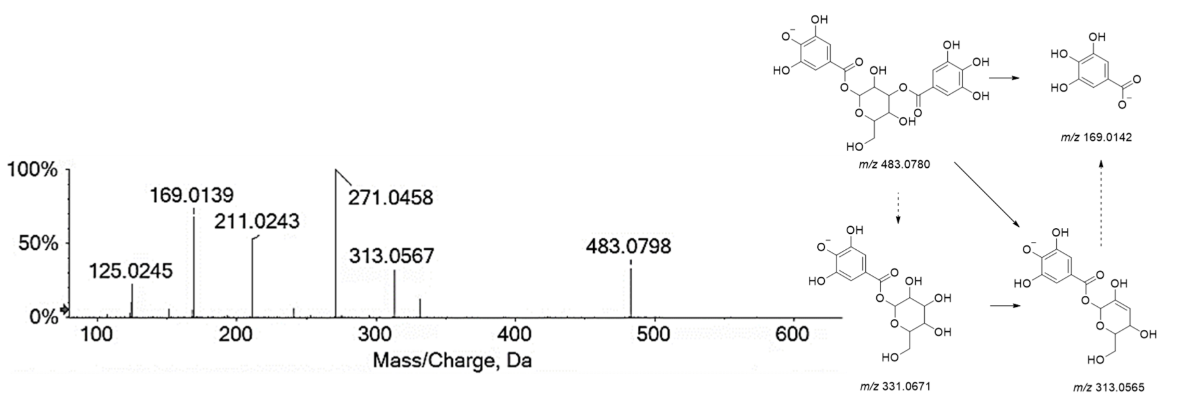

Figure S6. Digalloyl hexoses **(A) 7**, and **(B) 30** TOF-MS/MS spectra and their putative fragmentation patterns; theoretical mass is reported under each structure.

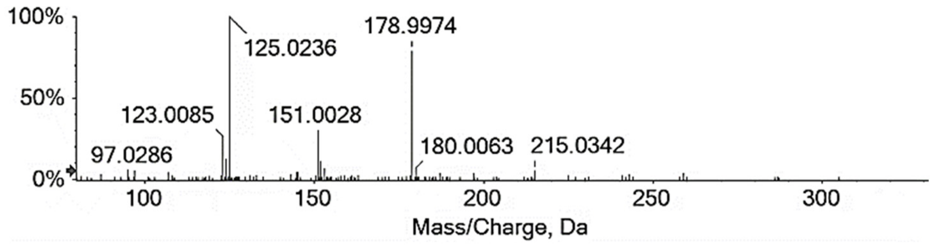

Figure S7. TOF-MS/MS spectra of compound **17**, tentatively identified as galloyl dihydroxybenzoic acid.

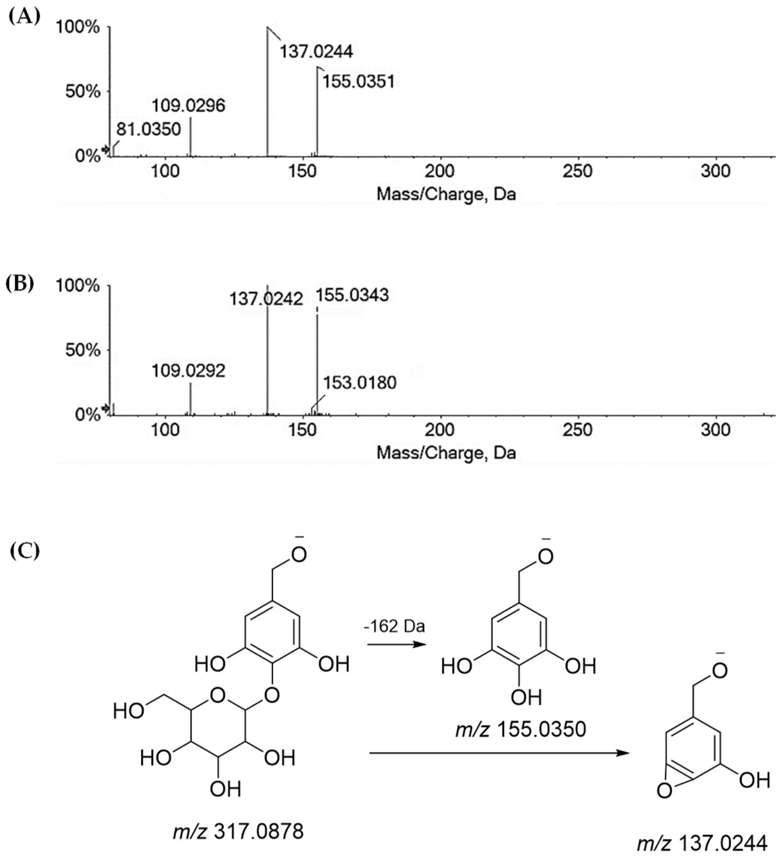

Figure S8. TOF-MS/MS spectra of compounds **8 (A)** and **11 (B)**. In panel **(C)** the putative fragmentation pattern of compound **8** is depicted.

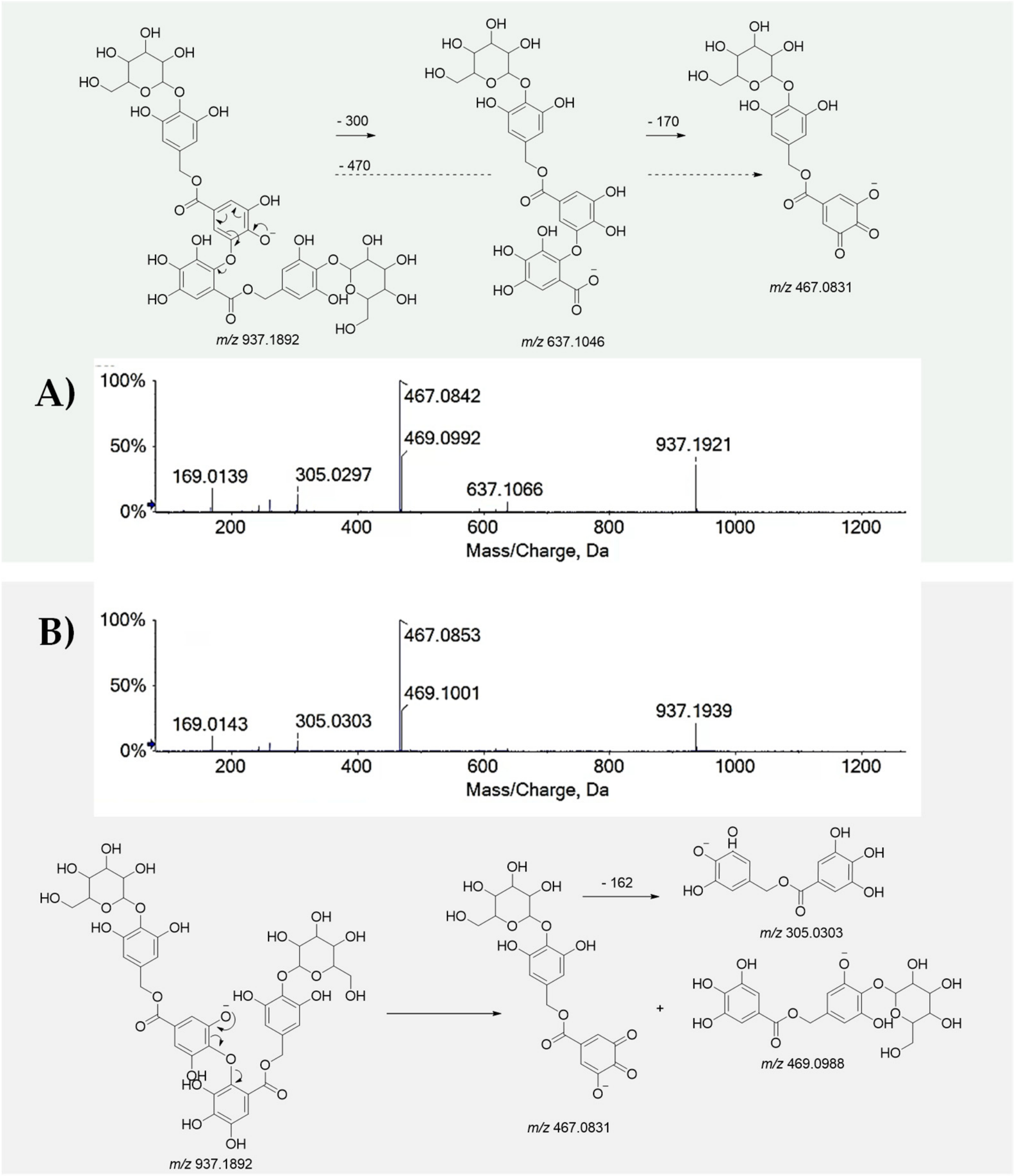

Figure S9. TOF-MS/MS spectra of **(A)** chestanin **(49)** and **(B)** isochestanin **(58).** Putative fragmentation patterns are depicted; theoretical mass is reported under each structure.

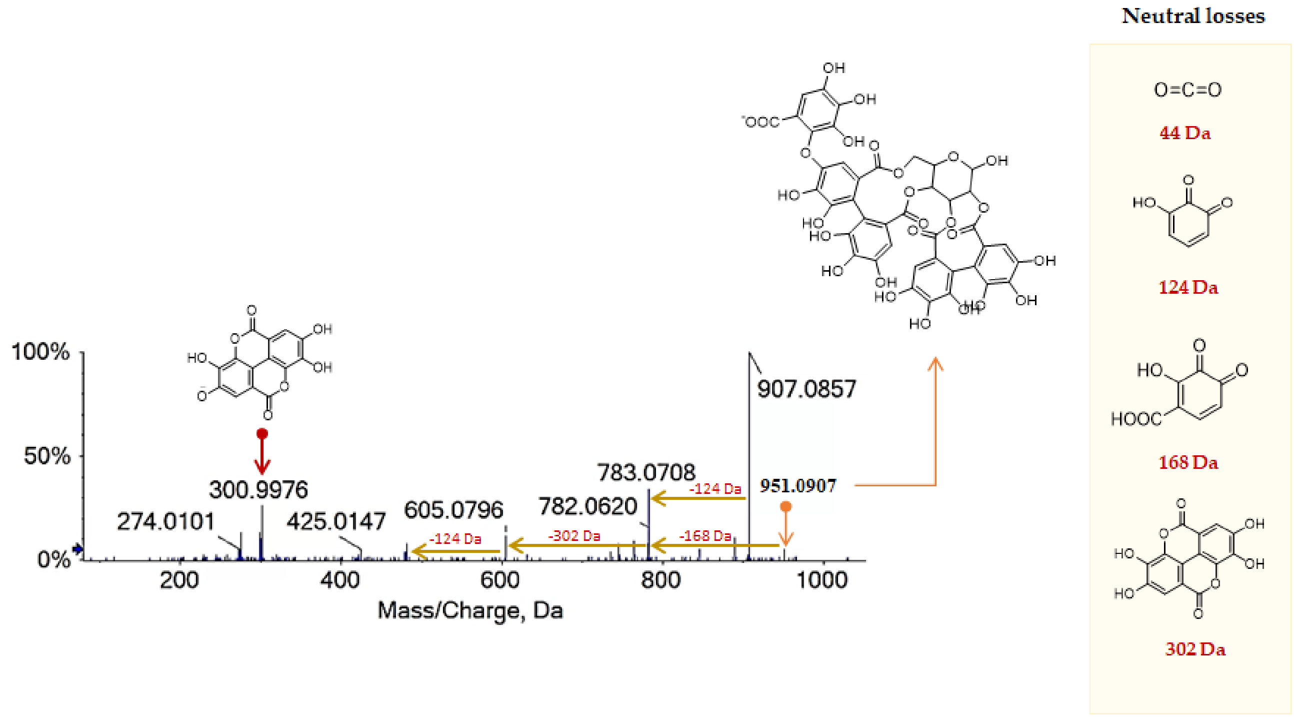

Figure S10. TOF-MS/MS spectra of compound **23** with $[M-H]^-$ ion at *m*/*z* 951.0907. Structures of observed neutral losses are depicted.

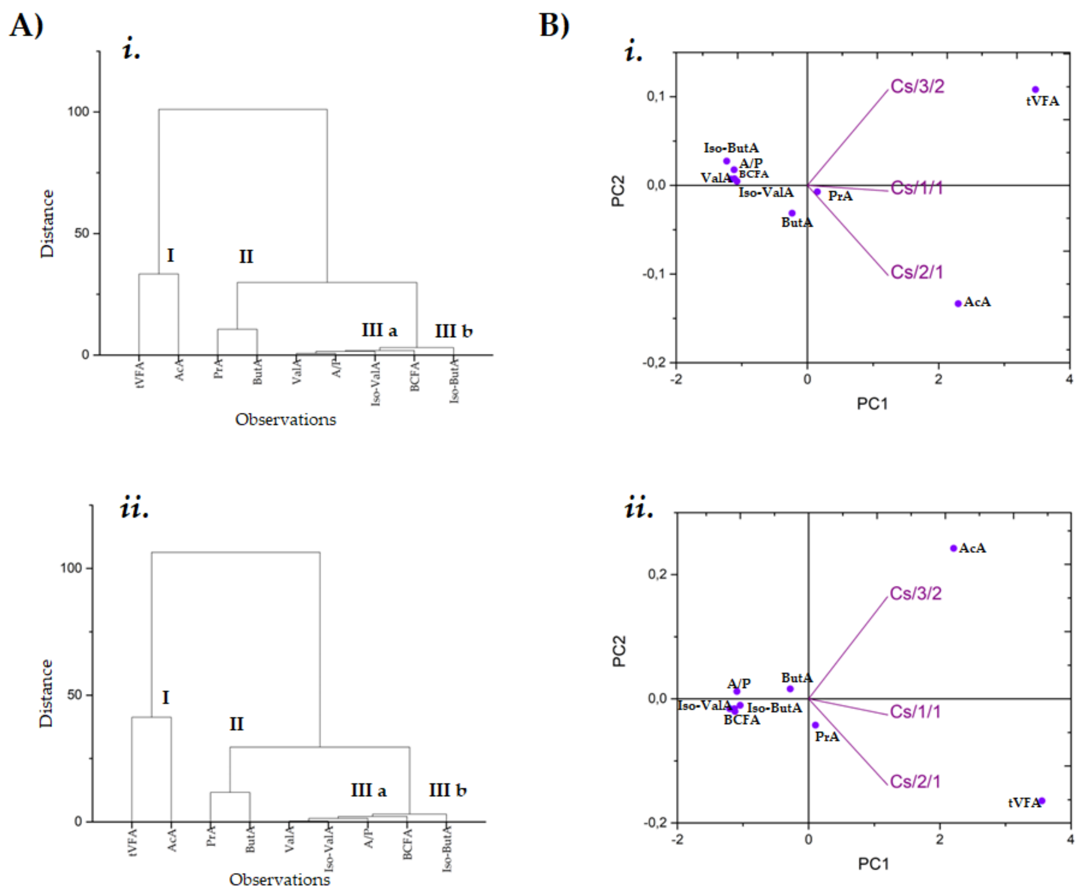

Figure S11. (**A**) Dendrograms of different volatile fatty acids obtained by treatment at 50 (***i.***) and 200 mg (***ii.***); (**B**) (***i.***) PCA (% variance on PC1 99.8; on PC2 0.2) of VFAs at 50 mg-dose level; (***ii.***) PCA (% variance on PC1 99.6; on PC2 0.4) of VFAs 200 of Cs/1/1, Cs/2/1 and Cs/3/2 fractions.

Table S1. Compounds tentatively identified in the chestnut Cs/1/1 alcoholic extract and its Cs/2/1 and Cs/3/2 fractions. Rt = retention time; RDB = ring double bond equivalent value. Base peak fragments are reported in bold.

Peak	Rt	Tentative assignment	Formula	$[M-H]^-$ found (*m/z*)	error (ppm)	RDB	MS/MS fragment ions (*m/z*) and relative intensity
**1**	0.288	Quinic acid	$C_7H_{12}O_6$	191.0562	0.5	2	191.0543; 127.0296; 93.0343; 87.0086; **85.0295**
**2**	0.602	Galloyl hexose I	$C_{13}H_{16}O_{10}$	331.0670	-0.2	6	331.0675; 271.0452; 211.0246; 169.0140; 151.0030; **125.0243**; 124.0164; 107.0136
**3**	0.680	Pyrogallol gallic acid	$C_{13}H_{10}O_8$	293.0302	0.1	9	169.0130; 168.0056; **125.0238**; 124.0161; 123.0081; 95.0132
**4**	0.680	Hexahydroxydiphenic acid	$C_{14}H_{10}O_{10}$	337.0204	0.8	10	169.0142; **125.0243**; 124.0163; 95.0137
**5**	0.721	Gallic acid	$C_7H_6O_5$	169.0151	5.0	5	**125.0239**; 124.0159
**6**	0.776	Galloyl hexose II	$C_{13}H_{16}O_{10}$	331.0669	-0.5	6	331.0651; 271.0461; 211.0242; 169.0142; 151.0034; **125.0242**; 124.0164; 107.0138
**7**	1.008	Digalloyl hexose	$C_{20}H_{20}O_{14}$	483.0785	1.0	11	483.0776; 331.0673; 313.0544; 211.0249; **169.0132**; 125.0241
**8**	1.124	3,4,5-trihydroxybenzyl hexoside I (e.g. crenatin)	$C_{13}H_{18}O_9$	317.0879	0.3	5	155.0340; **137.0242;** 109.0293
**9**	1.163	NHTP-HHDP-hexose I (e.g.,castalagin/vescalagin)	$C_{41}H_{26}O_{26}$	933.0640; 466.0328 $[M-2H]^{2-}$	1.8	29	**933.0680;** 915.0558; 871.0690; 631.0606; 569.0601; 467.0282; 300.9986
**10**	1.260	Dihydroxybenzoic acid hexoside	$C_{13}H_{16}O_9$	315.0724	0.8	6	152.0111; 109.0293; **108.0219**
**11**	1.417	3,4,5-trihydroxybenzyl hexoside II	$C_{13}H_{18}O_9$	317.0877	-0.3	5	155.0343; **137.0242**; 109.0292
**12**	1.535	Galloyl hexose III	$C_{13}H_{16}O_{10}$	331.0674	1.0	6	169.0142; **125.0244**
**13**	1.632	Bis-HHDP-hexose I (e.g.,pedunculagin/casuariin)	$C_{34}H_{24}O_{22}$	783.0700	1.7	23	783.0735; 481.0643; **300.9993**; 275.0199
**14**	1.768	Galloyl shikimic acid	$C_{14}H_{14}O_9$	325.0554	-3.4	8	325.0520; **169.0132**; 125.0273;124.0161
**15**	1.827	NHTP-HHDP-hexose II (e.g.,castalagin/vescalagin)	$C_{41}H_{26}O_{26}$	933.0641; 466.0326 $[M-2H]^{2-}$	0.2	29	613.0440; 467.0292; **300.9996**; 299.9908; 275.0205; 257.0080; 229.0135; 169.0138
**16**	2.087	Galloyl shikimic acid	$C_{14}H_{14}O_9$	325.0563	-0.6	8	271.1001; 169.0130; **125.0235**; 124.0159; 93.0351
**17**	2.187	Galloyl dihydroxybenzoic acid	$C_{14}H_{10}O_8$	305.0305	0.7	10	178.9974; 151.0028; **125.0236**
**18**	2.385	Galloyl shikimic acid	$C_{14}H_{14}O_9$	325.0566	-0.1	8	325.0532; 169.0141; **125.0237**; 124.0265; 107.0138
**19**	3.314	Bis-HHDP-hexose II (e.g.,pedunculagin/casuariin)	$C_{34}H_{24}O_{22}$	783.0696	1.2	23	783.0720; 481.0624; **300.9984**; 275.0188; 249.0396; 169.013
**20**	3.929	*p*-Coumaric acid hexoside I	$C_{15}H_{18}O_8$	325.0926	-0.9	7	163.0470; **119.0481**
**21**	4.589	Galloyl-chebuloyl-HHDP-hexose I (e.g., chebulagic acid)	$C_{41}H_{30}O_{27}$	953.0901	-0.1	27	953.0915; 909.1024; **785.0858**; 765.0585; 425.0136; 300.9979; 299.9902; 275.0186
**22**	4.609	*p*-Coumaric acid hexoside II	$C_{15}H_{18}O_8$	325.0926	-0.9	7	163.0390; **119.0502**
**23**	4.966	HHDP-valoneoyl-hexose	$C_{41}H_{28}O_{27}$	951.0761	1.7	28	951.0875; **907.0857**; 889.0935; 783.0708; 605.0796; 481.0693; 425.0147; 300.9976
**24**	4.988	Caffeic acid hexoside	$C_{15}H_{18}O_9$	341.0871	-1.2	7	179.0329; **135.0447**
**25**	5.268	Digalloyl-HHDP-hexose I	$C_{34}H_{26}O_{22}$	785.0857	1.8	22	785.0889; 615.0645; 483.0799; 419.0620; **300.9987;** 275.0193; 249.0400
**26**	5.628	5-*O*-Caffeoyl quinic acid	$C_{16}H_{18}O_9$	353.0884	1.7	8	**191.0560**; 85.0299
**27**	6.004	Galloyl-HHDP-hexose	$C_{27}H_{22}O_{18}$	633.0742	1.8	17	633.0739; 463.0520; **300.9978**; 275.0179; 249.0408
**28**	6.004	Galloyl-bis-HHDP-hexose I (e.g., stachyurin/casuarinin)	$C_{41}H_{28}O_{26}$	935.0818	2.1	28	**935.0792**; 917.0989; 873.0778; 855.0683; 783.0718; 633.0721; 571.0715; 300.9969; 299.0169; 275.0179

**29**	6.244	Methylvaloneoyl–NHTP–hexose (e.g.,vescavaloninic/castavaloninic acid methyl ester)	$C_{49}H_{32}O_{31}$	557.0405 [M-2H]$^{2-}$	1.8	34	933.0706; 631.0572; 466.0307; 425.0188; 300.9994; 275.0215; **181.0134**; 153.0201
**30**	6.165	Digalloyl hexose	$C_{20}H_{20}O_{14}$	483.0788	1.6	11	483.0798; 331.0666; 313.0567; **271.0458;** 211.0243; 169.0139; 125.0245
**31**	6.560	Galloyl-bis-HHDP-hexose II (e.g., stachyurin/casuarinin)	$C_{41}H_{28}O_{26}$	935.0801	0.5	28	**935.0826**; 917.0730; 873.0830; 783.0713; 633.0740; 571.0753; 419.0628; 300.9979; 299.0187; 275.0184
**32**	6.500	Galloyl-chebuloyl-HHDP-hexose II (e.g., chebulagic acid)	$C_{41}H_{30}O_{27}$	953.0909	0.8	27	**953.0966**; 935.0828; 909.1031; 785.0875; 633.0771; 300.9982; 275.0194
**33**	6.546	Trigalloyl hexose I	$C_{27}H_{24}O_{18}$	635.0892	0.3	16	635.0875; 483.0810; 465.0678; **313.0540**; 169.0131
**34**	6.819	Chesnatin	$C_{27}H_{26}O_{18}$	637.1064	2.8	15	637.1036; 469.1002; **467.0857**; 305.0299; 260.0327; 169.0143; 166.9984
**35**	7.080	3-O-*p*-Coumaroyl quinic acid	$C_{16}H_{18}O_{8}$	337.0925	-1.2	8	**191.0561**; 173.0450; 119.0500; 93.0342
**36**	7.218	3-O-*p*-Coumaroyl quinic acid	$C_{16}H_{18}O_{8}$	337.0933	1.2	8	**191.0556**; 173.0450; 119.0503; 93.0345
**37**	7.297	Digalloyl-HHDP-hexose II	$C_{34}H_{26}O_{22}$	785.0856	1.7	22	785.0884; 633.0755; 483.0795; **300.9991**; 275.0196; 249.0403
**38**	7.417	Digalloyl deoxyhexose	$C_{20}H_{20}O_{13}$	467.0843	2.5	11	467.0840; 449.0731; **423.0933**; 315.0715; 297.0611; 169.0137; 152.0112; 125.0238
**39**	8.110	Isochesnatin	$C_{27}H_{26}O_{18}$	637.1063	2.6	15	593.1180; 469.1004; 293.0301; 261.0301; **169.0142**
**40**	8.327	Galloyl phenol hexoside I (e.g.,cretanin)	$C_{20}H_{22}O_{13}$	469.0996	1.8	10	**169.0150**; 125.0250
**41**	8.581	Galloyl-bis-HHDP-hexose III	$C_{41}H_{28}O_{26}$	935.0797; 467.0375 [M-2H]$^{2-}$	0.1	28	391.0301; **300.9982**; 299.9900; 275.0184; 169.0137
**42**	8.721	Galloyl phenol hexoside II	$C_{20}H_{22}O_{13}$	469.0997	2.0	10	**169.0142**; 125.0244
**43**	8.919	Trigalloyl hexose II	$C_{27}H_{24}O_{18}$	635.0905	2.4	16	635.0923; **483.0795**; 465.0689; 313.0571; 271.0450; 169.0140
**44**	9.000	Trigalloyl-valoneoyl-HHDP-hexose (e.g.,rugosin A)	$C_{48}H_{34}O_{31}$	552.0490 [M-2H]$^{2-}$	1.1	32	937.1942; 785.0853; 767.9712; 749.0615; 615.0636; 425.0152; **300.9987**; 299.9907; 169.0137; 125.0242
**45**	9.455	Trigalloyl-HHDP-hexose	$C_{41}H_{30}O_{26}$	937.0947; 468.0454 [M-2H]$^{2-}$	-0.6	27	767.0768; 615.0654; 463.0522; **300.9995**; 283.9963; 275.0199; 245.0086; 231.0301; 169.0149; 125.0253
**46**	9.595	Quercetin pentosyl-hexoside	$C_{26}H_{28}O_{16}$	595.1318	2.3	13	595.1314; 343.0462; 301.0346; **300.0268;** 271.0232
**47**	9.704	Tetragalloyl hexose	$C_{34}H_{28}O_{22}$	787.1007	1.0	21	787.1060; **635.0927**; 617.0824; 573.0914; 465.0691; 313.0555; 295.0452; 169.0141
**48**	9.704	Ellagic acid pentoside	$C_{19}H_{14}O_{12}$	433.0415	0.6	13	433.0398; 300.9979; **299.9902;** 244.0003
**49**	9.754	Chestanin	$C_{40}H_{42}O_{26}$	937.1894	0.3	20	937.1921; 637.1066; 469.0992; **467.0824**; 305.0297; 169.0139
**50**	9.969	Quercetin hexuronide I	$C_{21}H_{18}O_{13}$	477.0686	2.6	13	477.0687; **301.0356**; 283.0236; 255.0294; 227.0338; 151.0036
**51**	9.911	Quercetin 3-*O*-hexoside I	$C_{12}H_{20}O_{12}$	463.0893	2.4	12	463.0894; 301.0351; **300.0276**; 271.0243; 255.0295; 151.0033
**52**	10.009	Rutin	$C_{27}H_{30}O_{16}$	609.1479	2.9	13	609.1484; 301.0352; **300.0277**; 271.0244; 2550296
**53**	10.067	Quercetin 3-*O*-hexoside II	$C_{12}H_{20}O_{12}$	463.0892	2.2	12	463.0901; 301.0357; **300.0280**; 271.0252; 255.0299; 243.0298
**54**	10.315	Quercetin hexuronide II	$C_{21}H_{18}O_{13}$	477.0680	1.1	13	477.0687; **301.0356**; 283.0236; 255.0300; 227.0338; 151.0036
**55**	10.315	Quercetin 3-*O*-hexoside III	$C_{12}H_{20}O_{12}$	463.0890	1.7	12	463.0908; 301.0357; **300.0276**; 271.0246; 255.0296; 151.0033
**56**	10.379	Benzyl-dihydroxybenzoate-*O*-pentosil-hexoside I	$C_{25}H_{30}O_{13}$	537.1624	1.9	11	429.1045; 297.0591; 243.0656; **153.0198;** 135.0086
**57**	10.515	Quercetin 3-*O*-pentoside	$C_{20}H_{18}O_{11}$	433.0786	1.5	12	433.0773; 301.0346; **300.0270**; 271.0240; 255.0289
**58**	10.592	Isochestanin	$C_{40}H_{42}O_{26}$	937.1904	1.3	20	937.1939; 469.1001; **467.0853**; 303.0303; 169.0143
**59**	10.635	Benzyl-dihydroxybenzoate-*O*-hexoside	$C_{20}H_{22}O_{9}$	405.1192	0.2	10	405.1164; 297.0611; 243.0643; 153.0195; **135.0082**; 109.0284
**60**	10.810	Kaempferol deoxyhexosyl-hexoside I	$C_{27}H_{30}O_{15}$	593.1533	3.5	13	593.1545; **285.0405**; 284.0330; 255.0301
**61**	10.887	Isorhamnetin deoxyhexoside I	$C_{21}H_{20}O_{11}$	447.0941	1.8	12	447.0949; 315.0140; 301.0358; 300.0280; 285.0408; 284.0333; 271.0246; **255.0302**; 227.0350

**62**	11.099	Isorhamnetin deoxyhexosyl-hexoside I	$C_{28}H_{32}O_{16}$	623.1632	2.2	13	623.1650; **315.0513**: 314.0435; 300.0273; 299.0195; 271.0244
**63**	11.176	Kaempferol deoxyhexosyl-hexoside II	$C_{27}H_{30}O_{15}$	593.1526	2.4	13	593.1557; **285.0409**; 284.0328; 255.0299
**64**	11.196	Isorhamnetin 3-*O*-hexoside I	$C_{22}H_{22}O_{12}$	477.1046	1.6	12	477.1059; 315.0503; **314.0435**; 300.0270; 299.0187; 271.0248
**65**	11.293	Isorhamnetin deoxyhexoside II	$C_{21}H_{20}O_{11}$	447.0935	0.5	12	447.0932; 315.0142; 314.0065; 300.0266; 299.9910; 285.0394; 284.0319; 271.0240; **255.0296**; 227.0344
**66**	11.293	Isorhamnetin hexuronide	$C_{22}H_{20}O_{13}$	491.0841	2.0	13	491.0817; 315.0507; **300.0268**; 271,0237; 255.0290
**67**	11.510	Isorhamnetin deoxyhexosyl-hexoside II	$C_{28}H_{32}O_{16}$	623.1633	2.3	13	623.1647; **315.0508**: 314.0428; 300.0273; 299.0195; 271.0244
**68**	11.570	Isorhamnetin 3-*O*-hexoside II	$C_{22}H_{22}O_{12}$	477.1051	2.6	12	477.1049; 315.0495; **314.0422**; 300.0273; 299.0194; 285.0405; 271.0248
**69**	11.980	Trimethylellagic acid hexose	$C_{23}H_{24}O_{13}$	551.1057 $[M+FA]^-$	n.c.	n.c.	343.0457; **328.0216**; 312.9983; 297.9743
**70**	12.181	Kaempferol acetylhexoside I	$C_{23}H_{22}O_{12}$	489.1058	4.0	13	489.1065; 285.0399; **284.0325**; 255.0295; 227.0346
**71**	12.402	Quercetin	$C_{15}H_{10}O_7$	301.0354	-0.3	11	301.0348; 273.0396; 227.0344; **151.0031;** 107.0152
**72**	12.737	Luteolin	$C_{15}H_{10}O_6$	285.0398	-2.3	11	285.0394; 241.0495; 175.0400; **133.0297**
**73**	12.819	Kaempferol acetylhexoside II	$C_{23}H_{22}O_{12}$	489.1051	2.6	13	489.1072; 285.0400; **284.0331**; 255.0300; 227.0346
**74**	13.160	Benzyl-dihydroxybenzoate-*O*-pentosil-hexoside II	$C_{25}H_{30}O_{13}$	537.1635	4.0	11	537.1937; **243.0661**; 152.0110; 108.0214
**75**	13.729	Quercetin *p*-coumaroyl-hexoside	$C_{30}H_{26}O_{14}$	609.1258	1.3	18	609.1258; 463.0855; **301.0337**; 300.0256; 271.0243; 151.0034
**76**	13.749	Quercetin *p*-coumaroyl-deoxyhexosyl-hexoside I	$C_{36}H_{36}O_{18}$	755.1854	3.3	19	755.1878; 609.1496: 591.1388; **301.0350**; 300.0270; 271.0248
**77**	13.769	Kaempferol *p*-coumaroyl-hexoside I	$C_{30}H_{26}O_{13}$	593.1326	2.4	13	593.1316; 447.0942; 307.0810; **285.0396**; 284.0319; 255.0289
**78**	13.968	Quercetin hexuronide butyl-ester	$C_{25}H_{26}O_{14}$	533.1316	2.9	13	533.1324; 357.0983; 301.0350; **300.0278**; 271.0243; 255.0291
**79**	13.968	Quercetin *p*-coumaroyl- deoxyhexosil-hexoside II	$C_{36}H_{36}O_{18}$	755.1850	3.3	19	755.1866; 609.1486: 591.1374; **301.0353**; 300.0276; 285.0399; 271.0248; 255.0291
**80**	13.984	Kaempferol	$C_{15}H_{10}O_6$	285.0403	-0.6	11	**285.0401**; 255.0296; 227.0348; 107.0150
**81**	14.174	Trimethylellagic acid deoxyhexose	$C_{23}H_{22}O_{12}$	535.1106 $[M+FA]^-$	n.c.	n.c.	343.04600; **328.0226**; 312.9990; 269.9802
**82**	14.174	Trimethylellagic acid I	$C_{17}H_{12}O_8$	343.0455	-1.3	12	328.0242; 312.9971; **297.9752;** 269.9794
**83**	14.366	Kaempferol deoxyhexosyl-*p*-coumaroyl-hexoside I	$C_{36}H_{36}O_{17}$	739.1894	1.9	19	739.1915; 593.1530; 575.1424; 453.1399; **285.0401**; 284.0318
**84**	14.436	Traumatic acid	$C_{12}H_{20}O_4$	227.1291	1.0	3	**183.1388**
**85**	14.561	Kaempferol *p*-coumaroyl-hexoside II	$C_{30}H_{26}O_{13}$	593.1311	1.7	13	447.0943; **285.0404**; 284.0330; 255.0296
**86**	14.643	Kaempferol deoxyhexosyl-*p*-coumaroyl-hexoside II	$C_{36}H_{36}O_{17}$	739.1899	2.9	19	739.1955; 593.1567; 575.1453; 453.1426; **285.0417**; 284.0335
**87**	15.139	Trimethylellagic acid II	$C_{17}H_{12}O_8$	343.0459	-0.1	12	328.0222; 312.9985; **297.9753**; 269.9796
**88**	15.563	Kaempferol acetyl *p*-coumaroyl-hexoside	$C_{32}H_{28}O_{14}$	635.1432	4.0	19	635.1441; 489.1068; **285.0404**; 284.0324
**89**	15.799	Methylkaempferol	$C_{16}H_{12}O_6$	299.0559	-0.7	11	299.0550; **284.0320**; 256.0368; 227.0336; 133.0298
**90**	16.019	3,7,24-trihydroxy-cycloartene-28-oic acid hexoside	$C_{36}H_{58}O_{10}$	695.4037 $[M+FA]^-$	n.c.	8	695.4039; 649.3972; **487.3450**
**91**	17.088	Kaempferol di-*p*-coumaroyl hexoside I	$C_{39}H_{32}O_{15}$	739.1695	3.6	24	739.1724; 593.1351; 453.1211; 307.0823; **285.0409;** 284.0326
**92**	17.284	Kaempferol di-*p*-coumaroyl hexoside II	$C_{39}H_{32}O_{15}$	739.1694	3.3	24	739.1715; 593.1337; 453.1211; **285.0409**; 284.0323
**93**	17.313	Acacetin	$C_{16}H_{12}O_5$	283.0611	-0.3	11	283.0594; **268.0363**; 211.0385; 151.0027; 117.0342
**94**	17.343	3,7-dihydroxynorcycloartane 24,28-dioic acid (e.g., castaartancrenoic acid A)	$C_{27}H_{42}O_6$	461.2921	2.7	7	**461.2908**; 443.2804; 417.2991; 415.2841
**95**	17.856	Cycloartane-type triterpene	$C_{30}H_{50}O_6$	505.3544	1.9	6	**505.3553;** 487.3429; 469.3327; 459.2758; 443.3201
**96**	18.337	Kaempferol acetyl di-*p*-coumaroyl hexoside I	$C_{41}H_{34}O_{16}$	781.1792	2.3	25	781.1803; 635.1434; 495.1325; **285.0401;** 284.0320; 145.0287
**97**	18.594	Kaempferol acetyl di-*p*-coumaroyl hexoside II	$C_{41}H_{34}O_{16}$	781.1797	2.9	25	781.1797; 635.1417; 495.1289**; 285.0394;** 284.0312; 145.0293
**98**	18.752	Cycloartane-type triterpene	$C_{27}H_{42}O_5$	445.2956	-0.8	7	**445.2959**; 427.2842; 399.2893; 383.2949
**99**	19.845	Cycloartane-type triterpene	$C_{30}H_{48}O_5$	487.3435	1.2	7	**487.3439;** 469.3326

**100**	19.954	Kaempferol di-acetyl di-*p*-coumaroyl hexoside I	$C_{43}H_{36}O_{17}$	823.1879	-0.1	26	823.1903; 677.1529; 659.1410; 617.1293; 537.1411**; 285.0396**; 284.0313; 145.0294
**101**	20.044	Cycloartane-type triterpene	$C_{30}H_{48}O_5$	487.3437	1.6	7	**487.3435**; 469.3325; 441.3378; 383.2937
**102**	20.144	Kaempferol di-acetyl di-*p*-coumaroyl hexoside II	$C_{43}H_{36}O_{17}$	823.1885	0.6	26	823.1916; 677.1551; 617.1326; 557.1125; **285.0406;** 284.0325
**103**	20.256	Cycloartane-type triterpene	$C_{30}H_{48}O_5$	487.3438	1.8	7	**487.3444;** 469.3328
**104**	20.425	Pentacyclic triterpene	$C_{30}H_{48}O_6$	503.3383	1.0	7	503.3394**; 471.3130;** 469.3319; 453.3015
**105**	20.606	DGMG (18:3)	$C_{33}H_{56}O_{14}$	675.3601	0.5	6	415.1457; 397.1344; **277.2172**; 235.0804
**106**	20.721	9-oxooctadeca-10,12-dienoic acid	$C_{18}H_{32}O_3$	293.2123	0.3	4	293.2123; **275.2015**; 231.2077; 183.1384
**107**	20.721	Pentacyclic triterpene	$C_{30}H_{48}O_6$	503.3385	1.4	7	503.3399; 485.3282; 471.3130; 445.2957; 401.2708; 387.2543; 359.2230; **319.1914**
**108**	21.100	9-oxooctadeca-10,12,15 trienoic acid	$C_{18}H_{28}O_3$	291.1965	-0.2	5	**291.1948;** 273.1847; 247.2077; 211.1332; 195.1379
**109**	21.318	l-PA (18:3)	$C_{21}H_{37}O_7P$	431.2212	1.8	4	277.2166; **152.9959**
**110**	21.846	Pentacyclic triterpene	$C_{30}H_{48}O_4$	471.3501<br>517.3558<br>$[M+FA]^-$	4.5	7	**471.3513**
**111**	22.313	l-PA (16:0)	$C_{19}H_{39}O_7P$	409.2358	-0.6	1	409.2335; 255.2325; **152.9957**
**112**	22.513	Pentacyclic triterpene	$C_{30}H_{48}O_4$	471.3487	1.5	7	**471.3478**
**113**	22.654	Pentacyclic triterpene	$C_{30}H_{48}O_5$	487.3440	2.9	7	487.3430; 469.3330; 455.3172; 385.2745; **303.1957**
**114**	22.695	Pentacyclic triterpene	$C_{30}H_{48}O_4$	471.3480	0.1	7	**471.3480**
**115**	22.784	Pentacyclic triterpene	$C_{30}H_{48}O_5$	487.3433	0.8	7	487.3433; **455.3179**
**116**	22.975	Linolenic acid	$C_{18}H_{30}O_2$	277.2172	-0.4	4	**277.2137**
**117**	23.115	Pentacyclic triterpene	$C_{30}H_{48}O_4$	471.3483	0.6	7	**471.3482;** 453.3371; 425.3433; 407.3324
**118**	23.215	Pentacyclic triterpene (e.g. ursolic or oleanolic acid)	$C_{30}H_{48}O_3$	455.3537	1.4	7	**455.3552**
**119**	23.496	Linoleic acid	$C_{18}H_{32}O_2$	279.2324	-2.0	3	**279.2314**

Table S2. Effects of *C. sativa* L. extracts at different doses (50 and 200 mg) on fermentation end products after 24 h of incubation. Total VFA: total volatile fatty acids (acetate + propionate + butyrate + iso-butyrate + valerate + iso-valerate); AcA = acetic acid; PrA = propionic acid; ButA = Butyric acid; ValA = valeric acid; iso-ButA = iso-butyric acid; iso-ValA = iso-valeric acid; BCFA= branched chain fatty acids (iso-butyrate + iso-valerate/tVFA); A/P=Acetate/Propionate. Along the row * $p < 0.05$, ** $p < 0.01$ and *** $p < 0.001$; NS: not significant; MSE: mean square error.

	**Control diet**	**Cs/1/1**		**Cs/2/1**		**Cs/3/2**		**MSE**
		**50mg**	**200mg**	**50mg**	**200mg**	**50mg**	**200mg**	
**pH**	6.32	6.34^{NS}	6.24^{NS}	6.35^{NS}	6.34^{NS}	6.35^{NS}	6.71^{NS}	0.02
**Total VFA (mmol/L)**	41.6	55.0***	54.3***	47.4***	58.5***	49.5**	38.7***	0.74
**AcA (% VFA)**	62.4	56.2***	55.9***	59.7*	56.6***	64.7^{NS}	61.4^{NS}	0.58
**PrA (% VFA)**	17.6	21.3***	22.6***	17.7^{NS}	19.5*	17.1^{NS}	18.2^{NS}	0.23
**ButA (% VFA)**	13.9	19.6***	18.8***	19.7***	20.9***	15.6*	18.3***	0.16

**ValA (% VFA)**	3.70	1.97***	2.11***	1.79***	1.87***	1.52***	1.44***	0.02
**iso-ButA (% VFA)**	0.61	0.50^{NS}	0.24**	0.51^{NS}	0.50^{NS}	0.50^{NS}	0.28**	0.005
**iso-ValA (% VFA)**	1.67	0.44***	0.38***	0.51***	0.54***	0.45***	0.36***	0.001
**BCFA (%VFA)**	2.29	0.97***	0.62***	1.02***	1.04***	0.95***	0.64***	0.004
**A/P (%VFA)**	3.53	2.64***	2.47***	3.37^{NS}	2.90**	3.77^{NS}	3.37^{NS}	0.01

Table S3. Values of Pearson's coefficient correlation, between antiradical (DPPH$^{•}$, ABTS$^{•+}$) activities, reducing activity (PFRAP), total phenol content (TPC), total flavonoid content (TFC) with fermentation parameters at the dose level of 50 mg. tVFA: total volatile fatty acids; AcA = acetic acid; PrA = propionic acid; ButA = Butyric acid; ValA = valeric acid; iso-ButA = iso-butyric acid; iso-ValA = iso-valeric acid; BCFA: branched chain fatty acids (iso-butyrate + iso-valerate/tVFA); A/P=Acetate/Propionate; OMD: organic matter degradability; OMCV: cumulative volume of gas related to incubated organic matter. R_{max}: maximum fermentation rate; T_{max}: time at which R_{max} occurs.

	TFC	*TPC*	*ABTS*	*DPPH*	*FRAP*	*pH*	*tVFA*	*AcA*	*PrA*	*ButA*	*ValA*	*iso-ButA*	*iso-ValA*	*BCFA*	*A/P*	*OMD*	*OMCV*	R_{max}	T_{max}
TFC	1,000																		
TPC	0,935	1,000																	
ABTS	0,985	0,982	1,000																
DPPH	0,968	0,994	0,997	1,000															
FRAP	0,927	1,000	0,978	0,991	1,000														
pH	-0,949	-0,999	-0,989	-0,998	-0,998	1,000													
tVFA	0,998	0,913	0,973	0,952	0,904	-0,929	1,000												
AcA	0,207	-0,153	0,036	-0,044	-0,174	0,111	0,263	1,000											
PrA	0,607	0,849	0,735	0,786	0,860	-0,826	0,560	-0,652	1,000										
ButA	-0,873	-0,989	-0,944	-0,967	-0,992	0,982	-0,843	0,297	-0,918	1,000									
ValA	-0,602	-0,280	-0,456	-0,384	-0,260	0,321	-0,648	-0,906	0,269	0,136	1,000								
Iso-ButA	0,608	0,287	0,462	0,390	0,266	-0,327	0,653	0,903	-0,262	-0,143	-1,000	1,000							
Iso-ValA	-0,677	-0,894	-0,794	-0,840	-0,903	0,874	-0,634	0,579	-0,996	0,950	-0,179	0,172	1,000						
BCFA	-0,383	-0,686	-0,536	-0,602	-0,701	0,654	-0,329	0,825	-0,967	0,785	-0,507	0,501	0,939	1,000					
A/P	-0,473	-0,755	-0,618	-0,678	-0,768	0,726	-0,421	0,764	-0,987	0,843	-0,418	0,412	0,969	0,995	1,000				
OMD	0,888	0,993	0,954	0,975	0,996	-0,987	0,860	-0,267	0,905	-1,000	-0,167	0,174	-0,940	-0,765	-0,826	1,000			
OMCV	0,979	0,844	0,930	0,898	0,832	-0,866	0,989	0,401	0,434	-0,756	-0,751	0,756	-0,514	-0,188	-0,285	0,776	1,000		
R_{max}	-0,914	-0,998	-0,970	-0,986	-0,999	0,995	-0,889	0,207	-0,877	0,996	0,227	-0,234	0,917	0,724	0,790	-0,998	-0,813	1,000	
T_{max}	0,578	0,251	0,429	0,356	0,230	-0,292	0,624	0,918	-0,298	-0,106	-1,000	0,999	0,209	0,533	0,446	0,137	0,731	-0,197	1,000

Table S4. Values of Pearson's coefficient correlation, between antiradical (DPPH•, ABTS•+) activities, reducing activity (PFRAP), total phenol content (TPC), total flavonoid content (TFC) with fermentation parameters at the dose level of 200 mg. tVFA: total volatile fatty acids; AcA = acetic acid; PrA = propionic acid; ButA = Butyric acid; ValA = valeric acid; iso-ButA = iso-butyric acid; iso-ValA = iso-valeric acid; BCFA: branched chain fatty acids (iso-butyrate + iso-valerate/tVFA); A/P=Acetate/Propionate; OMD: organic matter degradability; OMCV: cumulative volume of gas related to incubated organic matter. R_{max}: maximum fermentation rate; T_{max}: time at which R_{max} occurs.

TFC *TPC* *ABTS* *DPPH* *FRAP* *pH* *tVFA* *AcA* *PrA* *ButA* *ValA* *iso-ButA* *iso-ValA* *BCFA* *A/P* *OMD* *OMCV* R_{max} T_{max}

TFC	1,00																		
TPC	0,935	1,000																	
ABTS	0,985	0,982	1,000																
DPPH	0,968	0,994	0,997	1,000															
FRAP	0,927	1,000	0,978	0,991	1,000														
pH	-0,340	-0,651	-0,497	-0,564	-0,667	1,000													
tVFA	-0,951	-0,779	-0,884	-0,844	-0,766	0,033	1,000												
AcA	0,765	0,944	0,864	0,902	0,950	-0,866	-0,528	1,000											
PrA	-0,512	-0,783	-0,652	-0,710	-0,796	0,982	0,221	-0,945	1,000										
ButA	-0,488	-0,147	-0,331	-0,255	-0,126	-0,655	0,734	0,189	-0,500	1,000									
ValA	-0,895	-0,995	-0,959	-0,978	-0,997	0,723	0,714	-0,972	0,841	0,049	1,000								
iso-ButA	-0,949	-0,776	-0,881	-0,840	-0,762	0,027	1,000	-0,523	0,215	0,738	0,710	1,000							
iso-ValA	-0,537	-0,801	-0,674	-0,731	-0,814	0,976	0,249	-0,954	1,000	-0,474	0,856	0,244	1,000						
BCFA	-0,272	-0,595	-0,433	-0,503	-0,612	0,997	-0,039	-0,828	0,966	-0,707	0,672	-0,045	0,958	1,000					
A/P	0,652	0,878	0,773	0,821	0,888	-0,935	-0,385	0,987	-0,985	0,344	-0,921	-0,380	-0,990	-0,907	1,000				
OMD	0,521	0,185	0,367	0,292	0,164	0,625	-0,760	-0,151	0,466	-0,999	-0,087	-0,763	0,440	0,680	-0,307	1,000			
OMCV	0,603	0,281	0,457	0,385	0,261	0,545	-0,820	-0,052	0,376	-0,991	-0,185	-0,824	0,349	0,604	-0,211	0,995	1,000		
R_{max}	-0,550	-0,810	-0,686	-0,741	-0,823	0,972	0,265	-0,959	0,999	-0,460	0,864	0,259	1,000	0,953	-0,992	0,426	0,334	1,000	
T_{max}	0,966	0,995	0,996	1,000	0,993	-0,573	-0,838	0,906	-0,717	-0,245	-0,980	-0,835	-0,738	-0,512	0,827	0,282	0,375	-0,748	1,000
